# Hepcidin sustains Kupffer cell immune defense against bloodstream bacterial infection via gut-derived metabolites in mice

**DOI:** 10.1172/JCI189607

**Published:** 2025-07-03

**Authors:** Yihang Pan, Lihua Shen, Zehua Wu, Xueke Wang, Xiwang Liu, Yan Zhang, Qinyu Luo, Sijin Liu, Xiangming Fang, Qiang Shu, Qixing Chen

**Affiliations:** 1 Department of Clinical Research Center,; 2 Department of Gastroenterology, and; 3 Department of Cardiac Surgery, Children’s Hospital, Zhejiang University School of Medicine, National Clinical Research Center for Child Health, Hangzhou, China.; 4 State Key Laboratory of Environmental Chemistry and Ecotoxicology, Research Center for Eco-Environmental Sciences, Chinese Academy of Sciences, Beijing, China.; 5 Department of Anesthesiology, The First Affiliated Hospital, Zhejiang University School of Medicine, Hangzhou, China.

**Keywords:** Immunology, Infectious disease, Bacterial infections

## Abstract

Bloodstream bacterial infections cause one-third of deaths from bacterial infections, and eradication of circulating bacteria is essential to prevent disseminated infections. Here, we found that hepcidin, the master regulator of systemic iron homeostasis, affected Kupffer cell (KC) immune defense against bloodstream bacterial infections by modulating the gut commensal bacteria–derived tryptophan derivative indole-3-propionic acid (IPA). Hepcidin deficiency impaired bacterial capture by KCs and exacerbated systemic bacterial dissemination through morphological changes in KCs. Gut microbiota depletion and fecal microbiota transplantation revealed that the gut microbiota mediated the alteration of KCs volume. Mechanistically, hepcidin deficiency led to a decreased abundance of the IPA-producing commensal *Lactobacillus intestinalis* and a concomitant reduction in the gut-to-liver shuttling of its metabolite IPA. IPA supplementation or *L*. *intestinalis* colonization restored the KC volume and hepatic immune defense against bloodstream bacterial infection in hepcidin-deficient mice. Moreover, hepcidin levels in patients with bacteremia were associated with days of antibiotic usage and hospitalization. Collectively, our findings highlight a previously unappreciated role of hepcidin in sustaining KC-mediated hepatic defense against bloodstream bacterial infections through the gut commensal *L*. *intestinalis* and its tryptophan derivative IPA. More importantly, we show that restoring the crosstalk between the gut microbiota and liver through IPA-inspired therapies may offer a promising strategy for enhancing the host defense against bloodstream bacterial infections in those with low hepcidin levels and a high risk for bacterial infections.

## Abstract 2

Hepcidin sustains KC immune defense against bloodstream bacterial infections by modulating the gut commensal bacteria-derived tryptophan derivative indole-3-propionic acid.

## Graphical abstract

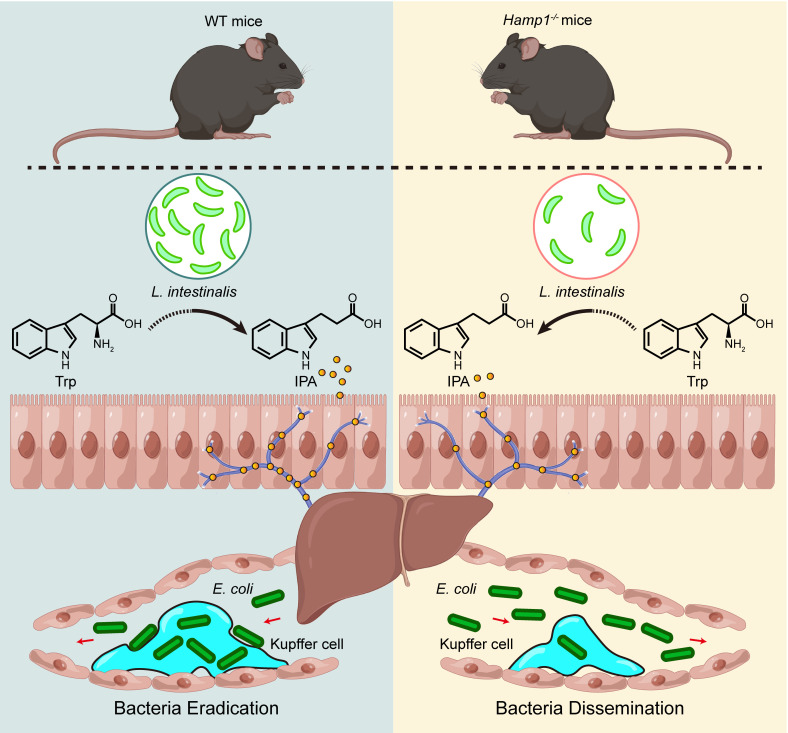

## Introduction

Bloodstream infection (BSI) remains a major cause of morbidity and mortality from infectious diseases worldwide, despite advances in critical care and treatment strategies and understanding of its pathophysiology (1). BSIs can present as primary infections caused by surgical intervention, the use of intravenous catheters, etc., or as secondary complications of infections elsewhere. Overgrowth and dissemination of invading pathogens in the circulation exacerbate collateral organ damage and usually lead to more severe conditions, such as infectious endocarditis, meningitis, sepsis, or septic shock. BSI confers an attributable mortality risk ranging from 7% to 35% and accounts for one-third of deaths from bacterial infections (2). Thus, rapid and effective removal of circulating bacteria is especially pivotal for preventing lethal consequences during BSI.

The liver filters one-third of the body’s total blood volume per minute and is a primary surveillance organ for intravascular infections (3), sequestering more than 90% of bacteria within 10 minutes after intravenous invasion (4, 5). As liver-resident macrophages, Kupffer cells (KCs) form a unique immune firewall within the vascular space of the hepatic sinusoids with a fundamental role in the clearance of blood-borne bacteria, a process that is crucial for preventing pathogen dissemination (6–9). The ability of KCs to capture and kill invading bacteria is well documented (9, 10), and the synergistic role of KCs with other immune cells in anti-infection defense studies has also been largely investigated (11, 12). However, little is known about the mechanisms that modulate the KC-mediated eradication of circulating bacteria.

Hepcidin is a master regulator of systemic iron metabolism that is produced predominantly by hepatocytes. Hepcidin controls iron concentrations in the extracellular fluid by binding ferroportin, which is the sole known cellular iron exporter, and inducing its degradation. As an acute-phase protein, hepcidin plays a critical role in host defense against bacterial infection (13). Hepcidin not only has direct bactericidal activity in vitro at nonphysiological high concentrations but also responds to bacterial infections through the iron deprivation of pathogenic microorganisms by regulating the intracellular/extracellular iron status to induce hypoferremia (14–16). Notably, low hepcidin levels are common in certain patients with hepatitis or cirrhosis, and these patients are more susceptible to severe bacteremia (17, 18). In mouse models of cecal ligation and puncture-induced polymicrobial sepsis, the knockdown of hepcidin in the liver led to the systemic spread of bacteria (19). This evidence highlights the importance of hepcidin in preventing systemic bacterial infections. However, the functional role of hepcidin in host defense is not fully understood, and the mechanisms by which hepcidin protects the host from systemic bacterial dissemination remain to be further explored.

Here, we explored the role of hepcidin in modulating hepatic immune defense against bloodstream bacterial infections. Using intravital imaging, we observed that hepcidin is essential for KC-mediated hepatic antibacterial defense in both gram-positive and -negative bacterial infection models. The combined strategies of gut microbiota depletion and fecal microbiota transplantation highlight the critical role of the gut microbiota in hepcidin-mediated regulation of KC function. We further explored the key commensal bacteria and their derived metabolites that participate in this process by fecal 16S rDNA sequencing and portal blood metabolomics analysis. We also investigated the correlation between hepcidin and the clinical status of infection in patients with bacteremia. Our data reveal the essential role of hepcidin in sustaining KC immune defense against bloodstream bacterial infections by modulating the gut–liver axis with the gut microbiota commensal bacteria *Lactobacillus intestinalis* and its tryptophan derivative indole-3-propionic acid (IPA).

## Results

### Hepcidin deficiency impairs hepatic immune defense against bloodstream bacterial infection.

**Figure 1:**
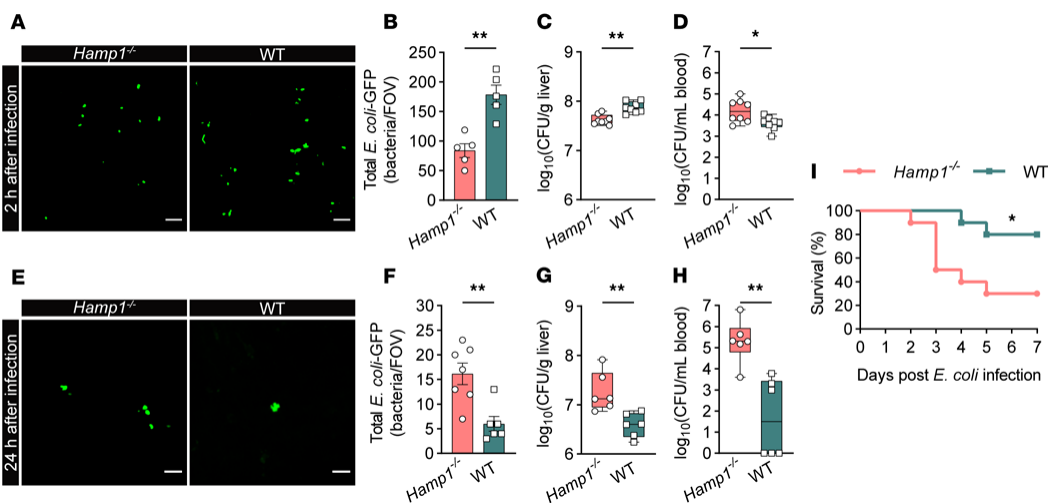
Hepcidin deficiency impairs hepatic immune defense against bloodstream bacterial infection. (**A**) Representative confocal intravital microscopy (IVM) images of the liver microcirculation of *Hamp1*^–/–^ and WT mice at 2 h after *E*. *coli*–GFP (green) infection. Scale bars: 20 μm. (**B**) Quantitative analysis of *E*. *coli*–GFP sequestered in the liver microcirculation (per field of view [FOV]) by confocal intravital microscopy. *n* = 5 per group; data are presented as mean ± SEM. (**C** and **D**) Bacterial load in the liver (**C**) and peripheral blood (**D**) at 2 h after *E*. *coli* infection in *Hamp1*^–/–^ and WT mice. *n* = 8 per group; data are presented as median ± interquartile range. (**E**) Representative IVM images of the liver microcirculation of *Hamp1*^–/–^ and WT mice at 24 h after *E*. *coli*–GFP infection. Scale bars: 20 μm. (**F**) Quantitative analysis of residual *E*. *coli*–GFP in the liver microcirculation (per FOV) at 24 h after *E*. *coli*–GFP infection by confocal intravital microscopy. *n* = 6–7 per group; data are presented as mean ± SEM. (**G** and **H**) Bacterial load in the liver (**G**) and peripheral blood (**H**) at 24 h after *E*. *coli* infection in *Hamp1*^–/–^ and WT mice. *n* = 6 per group; data are presented as median ± interquartile range. (**I**) Survival rate of *Hamp1*^–/–^ and WT mice after *E*. *coli* infection. *n* = 10 per group. *P < 0.05, **P < 0.01, by 2-tailed Student’s *t* test (**A**, **B**, **E**, and **F**), Mann-Whitney *U* test (**C**, **D**, **G**, and **H**), and Kaplan-Meier log-rank test (**I**). Data presented are from 5 (**A** and **B**), 8 (**C** and **D**), 4 (**E** and **F**), 3 (**G** and **H**), and 6 (**I**) independent experiments. Each symbol represents an individual mouse.

To test the hypothesis that hepcidin affects host defense against bloodstream bacterial infection, hepcidin-1 knockout (*Hamp1*^–/–^) and WT mice were intravenously infected with GFP-labeled *Escherichia coli* (*E*. *coli*–GFP), and circulating bacteria were rapidly sequestered by the liver of WT mice, but not *Hamp1*^–/–^ mice (Supplemental Video 1; supplemental material available online with this article; https://doi.org/10.1172/JCI189607DS1). Hepatic capture of *E*. *coli*–GFP was visualized via intravital confocal microscopy 2 hours after infection. Strikingly, circulating *E*. *coli*–GFP was efficiently sequestered in the livers of WT mice in a rapid fashion, whereas the livers of *Hamp1*^–/–^ mice were incapable of capturing bacteria (Figure 1, A and B). This inability to sequester bacteria was further supported by the significantly lower hepatic bacterial load and more pronounced bacteremia in *Hamp1*^–/–^ mice at 2 hours after infection with regular *E*. *coli* (Figure 1, C and D). However, the bacterial load in other vital organs, such as the lung, was not significantly different between the 2 groups of mice at the early stage after *E*. *coli* infection (Supplemental Figure 1A).

To investigate whether impaired hepatic bacterial capture affects bacterial clearance and leads to subsequent overwhelming systemic bacterial dissemination, mice infected with *E*. *coli*–GFP were imaged at 24 hours after infection to quantify the residual bacteria in the liver. *E*. *coli*–GFP almost completely disappeared from the livers of WT mice, whereas a considerable amount of *E*. *coli*–GFP persisted in the livers of *Hamp1*^–/–^ mice (Figure 1, E and F). Consistent with this finding, the livers of *Hamp1*^–/–^ mice presented a much greater bacterial load at 24 hours after infection with *E*. *coli* (Figure 1G). Moreover, *Hamp1*^–/–^ mice developed more severe bacteremia (Figure 1H) and exhibited markedly increased systemic dissemination of bacteria to other vital organs, such as the lungs, at 24 hours after infection (Supplemental Figure 1B). Thus, the failure of hepatic bacterial capture at the early stage after infection subsequently led to the systemic bacterial burden at the late stage after infection. Nevertheless, since iron is an essential nutrient for almost all living organisms, the effect of systemic high-iron status in *Hamp1*^–/–^ mice on bacterial replication also should not be overlooked (15, 20).

The impaired hepatic antibacterial defense of *Hamp1*^–/–^ mice resulted in a worse prognosis of bloodstream bacterial infection. Histopathologic analysis of the livers and lungs revealed that *Hamp1*^–/–^ mice experienced more severe tissue damage within 24 hours after infection (Supplemental Figure 1, C–F). More importantly, only 30% of *Hamp1*^–/–^ mice survived for 7 days after *E*. *coli* infection, whereas the survival rate was 80% in WT mice (Figure 1I). Together, these results suggest that hepcidin deficiency destroys hepatic immunity to capture and clear bloodstream bacteria and thus compromises the host defense against invading bacteria.

### Hepcidin deficiency impairs KC ability to clear invading bacteria.

We next sought to determine the mechanism underlying impaired hepatic antibacterial defense in *Hamp1*^–/–^ mice. As resident macrophages in the liver, KCs form a unique network with the vascular space of the liver sinusoids, acting as a “firewall” in detecting, capturing, and killing bloodstream pathogens to prevent disseminated infections (6–9, 21). The dominant role of KCs in hepatic immune defense against bacterial invasion has been extensively demonstrated by the depletion of KCs in such infectious models. We also observed that the absence of KCs with the use of liposome-encapsulated clodronate resulted in a dramatic impairment in the ability of the liver to sequester bacteria at 2 hours after *E*. *coli* infection (Supplemental Figure 2A), accompanied by extremely severe bacteremia (Supplemental Figure 2B). Not surprisingly, at 24 hours after infection, the hepatic bacterial load was lower in the mice treated with clodronate liposomes, while the blood bacterial load remained high because of the failure of hepatic bacterial capture in the early stage of infection (Supplemental Figure 2, C and D).

**Figure 2:**
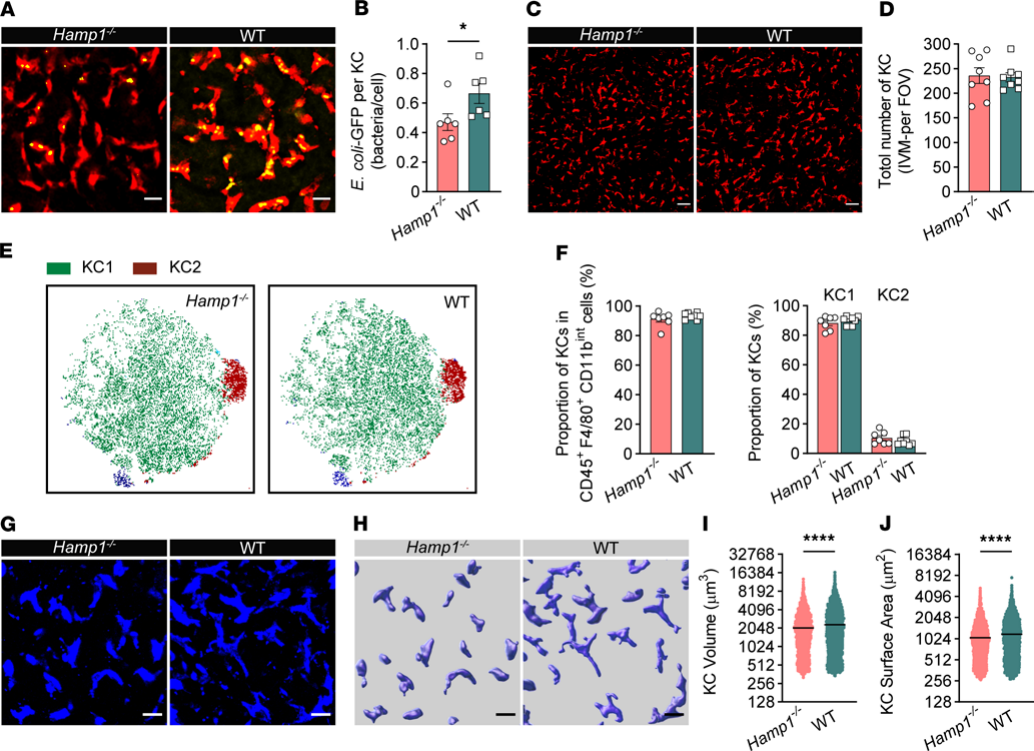
Hepcidin deficiency impairs KCs to clear invading bacteria. (**A**) Representative intravital microscopy (IVM) images showing KCs (red) capturing circulating *E*. *coli*–GFP (yellow) in *Hamp1*^–/–^ and WT mice. Scale bars: 20 μm. (**B**) Number of *E*. *coli*–GFP captured per KC. *n* = 6 per group; data are presented as mean ± SEM. (**C**) Representative IVM images of KCs (red) in *Hamp1*^–/–^ and WT mice. Scale bars: 50 μm. (**D**) Total number of KCs (per field of view) in *Hamp1*^–/–^ and WT mice. *n* = 8 per group; data are presented as mean ± SEM. (**E**) Flow cytometry analysis of liver CD45^+^F4/80^+^CD11b^int^ cells of *Hamp1*^–/–^ and WT mice with t-distributed stochastic neighbor embedding dimension reduction. (**F**) Quantitative analysis of subsets of KCs (KC1 and KC2) in liver CD45^+^F4/80^+^CD11b^int^ cells of *Hamp1*^–/–^ and WT mice. *n* = 7–8 per group; data are presented as mean ± SEM. (**G**–**J**) IVM images (**G**) combined with 3-dimensional reconstruction (**H**) to analyze KC volume (**I**) and surface area (**J**) in *Hamp1*^–/–^ and WT mice. Scale bars: 20 μm. *n* = 6 per group; data are presented as mean ± SEM. *P < 0.05, ****P < 0.0001, by 2-tailed Student’s *t* test. Data presented are from 6 (**A** and **B**), 8 (**C** and **D**), 4 (**E** and **F**), and 6 (**G**–**J**) independent experiments. Each symbol represents an individual mouse (**A**–**F**). Symbols represent individual KCs from 6 mice with 5 fields of view per mouse (**I** and **J**).

Next, we assessed the bacterial capture capacity of individual KCs in the liver via intravital confocal microscopy. Strikingly, compared with those of WT mice, the KCs of *Hamp1*^–/–^ mice captured less *E*. *coli*–GFP after BSI (Figure 2, A and B, and Supplemental Video 2). The inability to sequester bacteria was further confirmed by flow cytometry analysis, which revealed reduced *E*. *coli–*GFP uptake by KCs from *Hamp1*^–/–^ mice (Supplemental Figure 3, A and B).

Since bloodstream bacterial infections can be caused by both gram-negative and -positive bacteria, to extend the current findings, we further investigated the hepatic immune defense of *Hamp1*^–/–^ mice against BSI with the common clinical gram-positive bacterium *Staphylococcus aureus*. Similar to the *E*. *coli* infection model, at 2 hours after *S*. *aureus*-GFP infection, the invading bacteria were efficiently captured by KCs in WT mice. However, the KCs of *Hamp1*^–/–^ mice exhibited insufficient uptake of *S*. *aureus*-GFP (Supplemental Figure 4, A and B, and Supplemental Video 3). *Hamp1*^–/–^ mice also presented a lower hepatic bacterial load and more severe bacteremia when infected with *S*. *aureus* (Supplemental Figure 4, C and D). This compromised hepatic antibacterial defense led to the systemic retention of *S*. *aureus*, as evidenced by the greater residual *S*. *aureus*-GFP in the livers of *Hamp1*^–/–^ mice at 24 hours after infection (Supplemental Figure 4, E and F), as well as the greater bacterial burdens in the liver and peripheral blood during *S*. *aureus* infection (Supplemental Figure 4, G and H). Ultimately, all *Hamp1*^–/–^ mice died within 4 days, whereas 60.0% of the WT mice survived for 7 days after bloodstream *S*. *aureus* infection (Supplemental Figure 4I).

We then explored how KCs affect the hepatic antibacterial defense of *Hamp1*^–/–^ mice. We first examined whether there was a difference in KC number between *Hamp1*^–/–^ and WT mice. Both intravital confocal microscopy analysis (Figure 2, C and D) and immunofluorescence analysis (Supplemental Figure 5, A and B) revealed comparable total numbers of KCs between *Hamp1*^–/–^ and WT mice, suggesting that the impaired bacterial capture by KCs in *Hamp1*^–/–^ mice was not due to a reduction in the number of KCs.

Different phenotypes and subsets of KCs reportedly have distinct immune functions (22–24). Therefore, we next asked whether the distribution of KC subpopulations (23, 25) differed between *Hamp1*^–/–^ and WT mice. A t-distributed stochastic neighbor embedding dimension reduction analysis revealed that there was no difference in KC composition (CD45^+^F4/80^+^CD11b^int^MHCII^+^TIM4^+^ cells) between *Hamp1*^–/–^ and WT mice (Figure 2E). Moreover, the proportions of total KCs in CD45^+^F4/80^+^CD11b^int^ hepatic nonparenchymal cells, and KC subpopulations (identified as 2 clusters: KC1 characterized with CD45^+^F4/80^+^CD11b^int^MHCII^+^TIM4^+^ESAM^–^CD206^–^ cells and KC2 characterized with CD45^+^F4/80^+^CD11b^int^MHCII^+^TIM4^+^ESAM^+^CD206^+^ cells) in KCs were comparable (Figure 2F and Supplemental Figure 5C).

Studies have shown that the phagocytic function of KCs is also governed by their morphology, as their larger size facilitates an increased endovascular surface area for interaction with circulating pathogens (26, 27). Thus, we evaluated the cellular morphology of KCs in *Hamp1*^–/–^ and WT mice using intravital confocal microscopy combined with 3-dimensional reconstruction (Figure 2, G and H). Strikingly, the KCs of *Hamp1*^–/–^ mice generally had a much smaller volume and surface area than those of WT mice (Figure 2, I and J), which may account for the impaired hepatic bacterial capture capacity of *Hamp1*^–/–^ mice during bloodstream bacterial infections.

Efficient capture and successful eradication of invading bacteria are both important for the immune defense of KCs against bloodstream bacterial infection. Thus, we next investigated whether, in addition to phagocytosis, the intracellular bacterial killing ability of KCs was also affected in *Hamp1*^–/–^ mice. Mechanistically, the intracellular killing of bacteria by KCs mainly depends on 2 processes following phagocytosis: phagosome maturation with acidification and ROS generation (21). We examined phagosome acidification and ROS generation through the engulfment of pH- and ROS-sensitive reporter bacteria by KCs using intravital confocal microscopy, as previously described (28). However, there were no significant differences in either the acidification of *E*. *coli*–containing phagosomes (Supplemental Figure 6, A and B, and Supplemental Video 4) or the ROS generation capacity (Supplemental Figure 6, C and D, and Supplemental Video 5) between the KCs of *Hamp1*^–/–^ and WT mice.

To investigate the potential mechanisms that may associate with the functional changes, we performed RNA sequencing analysis of freshly isolated KCs from *Hamp1*^–/–^ and WT mice. Kyoto Encyclopedia of Genes and Genomes pathway enrichment analysis revealed that hepcidin deficiency caused differentially expressed genes highly involved in phagosome and lysosome functions as well as regulation of the actin cytoskeleton (Supplemental Figure 7A). Particularly, genes related to these biological processes, such as Toll-like receptor adaptor molecule 2, low-density lipoprotein receptor, and integrin α M, showed significantly downregulated expression in KCs from *Hamp1*^–/–^ mice (Supplemental Figure 7B). These results suggest that hepcidin deficiency impairs the phagocytic function of KCs, which contributes to the damped hepatic immune defense against BSI.

Taken together, these findings suggest that hepcidin deficiency impaired the bacterial capture ability but not the intracellular bacterial killing ability of KCs during bloodstream bacterial infections, which may be attributed to the morphological alterations of KCs under conditions of hepcidin deficiency.

### The gut microbiota mediates the malfunction of KC antibacterial defense in Hamp1^–/–^ mice.

**Figure 3:**
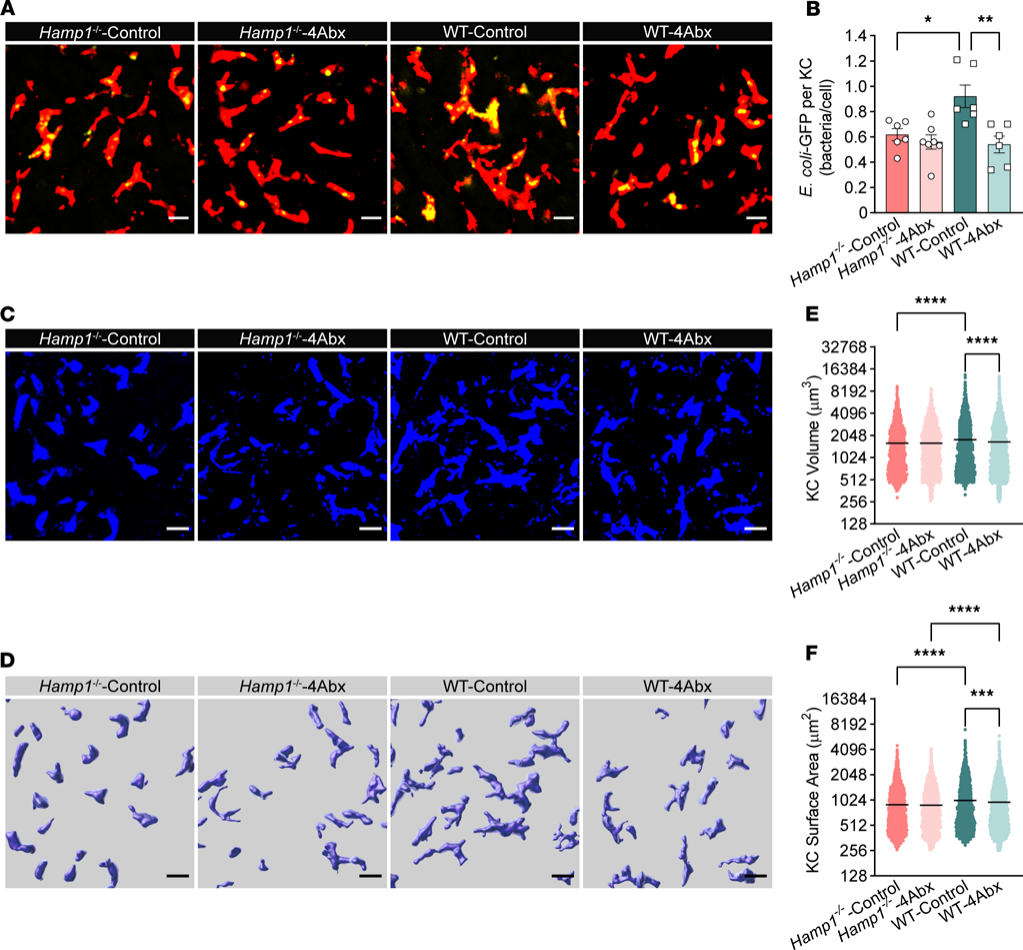
The gut microbiota mediates the malfunction of KC antibacterial defense in *Hamp1*^–/–^ mice. (**A**) Representative intravital microscopy (IVM) images showing KCs (red) capturing circulating *E*. *coli*–GFP (yellow) in *Hamp1*^–/–^ and WT mice treated with antibiotic cocktail (4Abx) or sterile water (control). Scale bars: 20 μm. (**B**) Number of *E*. *coli*–GFP captured per KC. *n* = 6–7 per group; data are presented as mean ± SEM. (**C**–**F**) IVM images (**C**) combined with 3-dimensional reconstruction (**D**) to analyze KC volume (**E**) and surface area (**F**) in *Hamp1*^–/–^ and WT mice treated with 4Abx or sterile water (control). Scale bars: 20 μm. *n* = 6–7 per group; data are presented as mean ± SEM. *P < 0.05, **P < 0.01, ***P < 0.001, ****P < 0.0001, by 1-way ANOVA followed by Šidák’s multiple-comparison test. Data presented are from 6 independent experiments (**A**–**F**). Each symbol represents an individual mouse (**A** and **B**). Symbols represent individual KCs from mice, and data are from 6–7 mice with 5 fields of view per mouse (**E** and **F**).

In addition to the hepatic artery, the liver’s blood supply is provided mainly by the portal vein, which collects venous blood that drains from the intestines (29). This anatomical connection allows the liver to communicate with the gut through the integration of signals composed of gut-derived nutrients, pathogens, microbiota, and microbial metabolites via the portal vein (30). The gut microbiota is a central element of the gut–liver axis and plays a pivotal role in shaping liver immune responses during health and disease (31, 32). We then asked whether the morphological and functional defects in KCs in *Hamp1*^–/–^ mice are associated with intestinal dysbiosis and concomitant alterations in signals in the gut–liver axis. We thus depleted the gut microbes in WT and *Hamp1*^–/–^mice with either an antibiotic cocktail (4Abx) or sterile water, followed by a 5-day washout period and subsequent bloodstream *E*. *coli*–GFP infection. Interestingly, hepatic intravital imaging revealed that, compared with sterile water–treated control mice, WT mice presented a markedly impaired capture capacity of KCs in those treated with antibiotics at the early stage after BSI. However, there were no significant differences in the capture capacity of KCs in *Hamp1*^–/–^ mice treated with antibiotics or sterile water (Figure 3, A and B, and Supplemental Video 6). We further observed the cellular morphology of the KCs (Figure 3, C and D). Consistent with the alterations in the bacterial capture capacity of KCs, gut microbiota depletion markedly reduced the surface area and volume of KCs in WT mice but did not affect the morphology of KCs in *Hamp1*^–/–^ mice (Figure 3, E and F). Notably, after antibiotic treatment, the volume of KCs in the 2 groups was comparable (Figure 3E), although the surface area of KCs in *Hamp1*^–/–^ mice was still smaller than that in WT mice (Figure 3F), which might be due to the critical role of the cellular volume in phagocytic efficiency, as phagocytosis depends on the formation of engulfment pseudopods surrounding target particles, and a larger cellular volume facilitates local expansion and retraction of engulfment pseudopods (33, 34). Moreover, bacterial loads of *Hamp1*^–/–^ and WT mice treated with antibiotics or sterile water were examined at 2 hours after *E*. *coli* infection. Treatment with antibiotics impaired hepatic bacterial capture and resulted in more severe bacteremia in WT mice but did not significantly alter bacterial loads in liver and peripheral blood of *Hamp1*^–/–^ mice (Supplemental Figure 8, A and B). These findings indicate that the gut microbiota is engaged in programming the KC defense against bloodstream bacterial infection and, more importantly, that hepcidin deficiency may lead to the loss of some intrinsic protective signals derived from the gut microbiota and thus compromise KC immune defense against invading bacteria.

**Figure 4:**
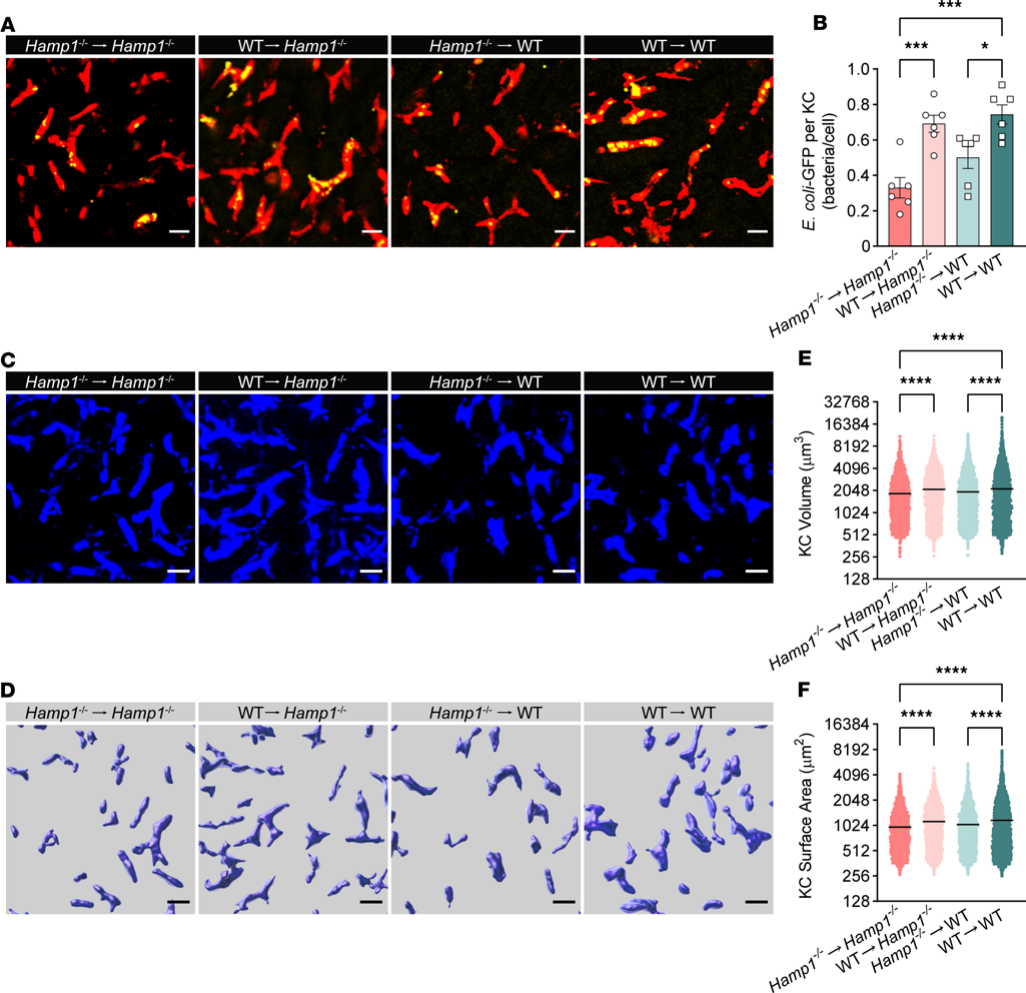
Fecal microbiota transplantation rescues malfunction of KC bacterial clearance in *Hamp1*^–/–^ mice. (**A**) Representative intravital microscopy (IVM) images showing KCs (red) capturing circulating *E*. *coli*–GFP (yellow) in *Hamp1*^–/–^ and WT mice that received fecal microbiota transplantation. Scale bars: 20 μm. (**B**) Number of *E*. *coli*–GFP captured per KC. *n* = 6 per group; data are presented as mean ± SEM. (**C**–**F**) IVM image (**C**) combined with 3-dimensional reconstruction (**D**) to analyze KC volume (**E**) and surface area (**F**) in *Hamp1*^–/–^ and WT mice that received fecal microbiota transplantation. Scale bars: 20 μm. *n* = 6 per group; data are presented as mean ± SEM. *P < 0.05, ***P < 0.001, ****P < 0.0001, by 1-way ANOVA followed by Šidák’s multiple-comparison test. Data presented are from 6 independent experiments (**A**–**F**). Each symbol represents an individual mouse (**B**), and symbols represent individual KCs from 6 mice with 5 fields of view per mouse (**E** and **F**).

To further define the role of the gut microbiota in the interaction between hepcidin and the antibacterial defense of KCs, we transplanted fecal microbiota from WT or *Hamp1*^–/–^ donors into antibiotic cocktail (4Abx)–pretreated WT or *Hamp1*^–/–^ recipients via oral gavage for 10 days before bloodstream *E*. *coli–*GFP infection. Interestingly, KCs from *Hamp1*^–/–^ recipients that received fecal microbiota from WT mice (WT→*Hamp1*^–/–^) exhibited improved bacterial capture capacity compared with those from *Hamp1*^–/–^ recipients (*Hamp1*^–/–^→*Hamp1*^–/–^). In contrast, KCs from WT recipients that received fecal microbiota from *Hamp1*^–/–^ donors (*Hamp1*^–/–^→WT) showed impaired bacterial capture capacity compared with those from WT recipients (WT→WT) (Figure 4, A and B, and Supplemental Video 7). Moreover, when KCs were imaged via intravital confocal microscopy and morphologically reconstructed (Figure 4, C and D), compared with fecal microbiota transplantation from *Hamp1*^–/–^ donors, fecal microbiota transplantation from WT donors markedly increased the surface area and volume of KCs in *Hamp1*^–/–^ recipients, whereas feces from *Hamp1*^–/–^ donors had a diametrically opposite effect on the morphology of KCs in WT recipients (Figure 4, E and F). These findings further emphasize the role of the gut microbiota in mediating the aberrant antibacterial defense of KCs induced by hepcidin deficiency.

### Hepcidin deficiency remaps portal blood metabolite profiles in mice.

The gut microbiota performs critical biological functions for the host by communicating with extraintestinal tissues or organs, primarily through the release of small-molecule metabolites (35, 36). Relying on the anatomical connection of the portal vein, the activity of KCs can be modulated by gut microbiota–derived metabolites (26, 30, 37).

**Figure 5:**
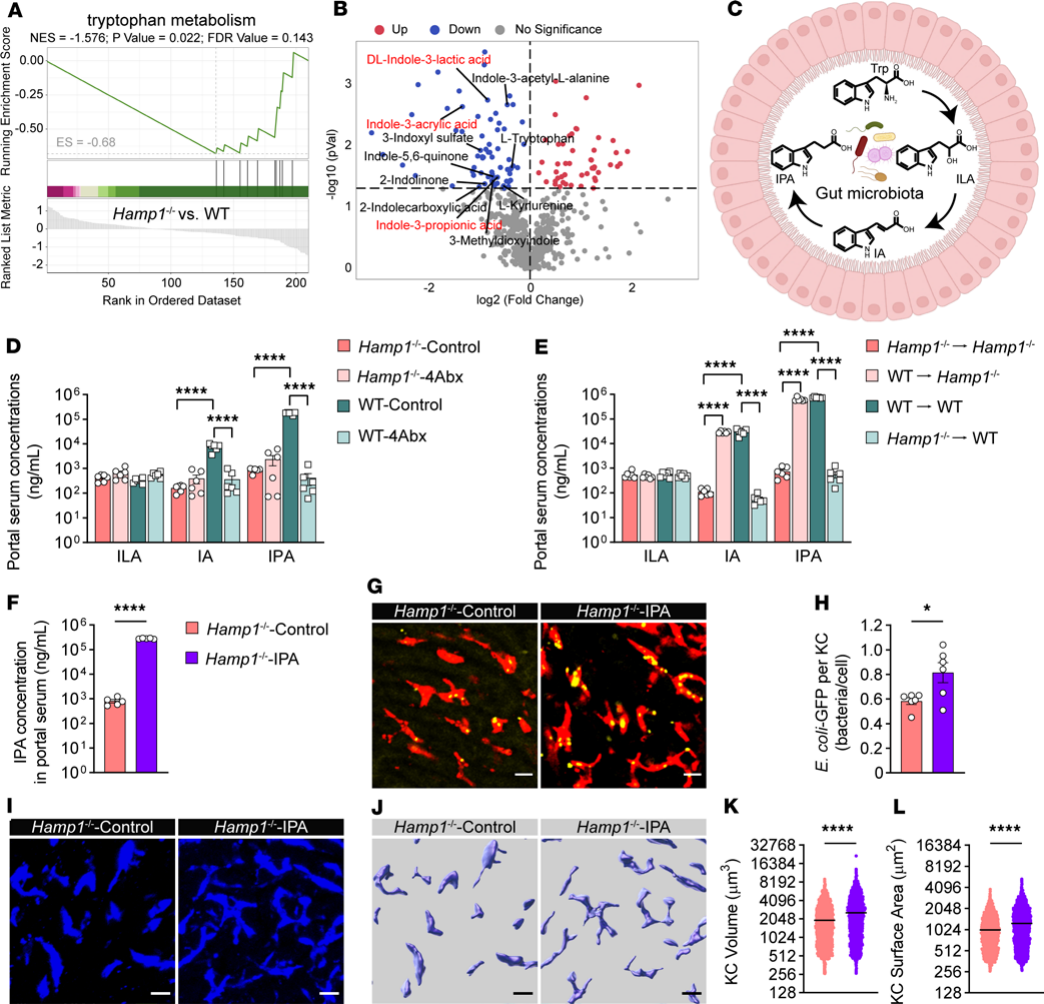
IPA supplementation restores hepatic immune defense against bloodstream bacterial infection in *Hamp1*^–/–^ mice. (**A**) Hepcidin deficiency downregulated metabolites involved in the tryptophan metabolism pathway. *n* = 4 per group. (**B**) Volcano plot showing the fold change and *P* value of gut microbiota–derived metabolites in portal blood from *Hamp1*^–/–^ and WT mice. *n* = 4 per group. (**C**) Diagram of tryptophan metabolized by the gut microbiota to ILA, IA, and IPA. (**D** and **E**) Portal blood levels of ILA, IA, and IPA in *Hamp1*^–/–^ and WT mice treated with either antibiotic cocktail (4Abx) or sterile water (control) (**D**) and in *Hamp1*^–/–^ and WT mice that received fecal microbiota transplantation (**E**). *n* = 6 per group; data are presented as mean ± SEM. (**F**) Portal blood level of IPA in *Hamp1*^–/–^ mice pretreated with IPA or sterile PBS. *n* = 5 per group; data are presented as mean ± SEM. (**G**) Representative intravital microscopy (IVM) images showing KCs (red) capturing circulating *E*. *coli*–GFP (yellow) in *Hamp1*^–/–^ mice pretreated with IPA or sterile PBS. Scale bars: 20 μm. (**H**) Number of *E*. *coli*–GFP captured per KC. *n* = 6 per group; data are presented as mean ± SEM. (**I**–**L**) IVM images (**I**) combined with 3-dimensional reconstruction (**J**) to analyze KC volume (**K**) and surface area (**L**) in *Hamp1*^–/–^ mice pretreated with IPA or sterile PBS. Scale bars: 20 μm. *n* = 6 per group; data are presented as mean ± SEM. *P < 0.05, ****P < 0.0001, by 1-way ANOVA followed by Šidák’s multiple-comparison test (**D** and **E**) or 2-tailed Student’s *t* test (**F**–**L**). Data presented are from 2 (**D** and **E**) and 4 (**G**–**L**) independent experiments. Each symbol represents an individual mouse (**D**–**H**). Symbols represent individual KCs from 6 mice with 5 fields of view per mouse (**K** and **L**). Trp, tryptophan.

We then performed untargeted metabolomics analysis using the sera of portal vein samples from *Hamp1*^–/–^ and WT mice. We first observed that the gross anatomy and microstructure of the intestine were comparable between *Hamp1*^–/–^ and WT mice (Supplemental Figure 9, A and B), indicating that hepcidin deficiency does not affect normal intestinal development or histomorphology. Partial least squares–discriminant analysis revealed distinct differences in metabolite composition between *Hamp1*^–/–^ and WT mice (Supplemental Figure 10A). We next scanned the portal blood metabolite panels that were significantly enriched between *Hamp1*^–/–^ and WT mice. Gene set enrichment analysis suggested that 1 interesting pathway, the tryptophan metabolism pathway, was significantly downregulated in *Hamp1*^–/–^ mice (Figure 5A and Supplemental Figure 10B). Notably, among the 68 significantly downregulated metabolites in *Hamp1*^–/–^ mice, the tryptophan derivatives dl-indole-3-lactic acid (ILA), indole-3-acrylic acid (IA), and IPA, which are sequentially metabolized from dietary tryptophan by the gut microbiota, have pronounced reduction simultaneously (Figure 5, B and C).

We then examined the concentrations of ILA, IA, and IPA in portal vein sera from *Hamp1*^–/–^ and WT mice gavaged with either an antibiotic cocktail (4Abx) or sterile water based on the initial screening for differentially abundant metabolites via untargeted metabolomics analysis to identify the differentially abundant metabolites that are critical for the immune functions of KCs disrupted by hepcidin deficiency. The portal vein level of ILA did not significantly differ among the groups (Figure 5D). The portal vein levels of the intermediate metabolite IA and the terminal product IPA were significantly lower in *Hamp1*^–/–^ mice gavaged with sterile water than in WT mice gavaged with sterile water. More importantly, in WT mice, the portal vein levels of IA and IPA were significantly lower in the antibiotic-treated group than in the water-treated control group. However, antibiotic treatment did not significantly alter the portal vein levels of IA and IPA in *Hamp1*^–/–^ mice.

We further measured portal vein serum levels of ILA, IA, and IPA in *Hamp1*^–/–^ and WT mice that received reciprocal fecal microbiota transplantation. The portal vein ILA level was not significantly altered among the groups regardless of the donor fecal microbiota (Figure 5E). Notably, *Hamp1*^–/–^ recipients who received fecal microbiota from WT donors (WT→*Hamp1*^–/–^) presented significantly greater portal blood IA levels and IPA levels than did those who received fecal microbiota from *Hamp1*^–/–^ donors (*Hamp1*^–/–^→*Hamp1*^–/–^), whereas WT recipients who received fecal microbiota from *Hamp1*^–/–^ donors (*Hamp1*^–/–^→WT) presented lower portal vein IA levels and IPA levels than did those who received fecal microbiota from WT mice (WT→WT). The changes in the concentrations of these tryptophan derivatives in the portal vein across different treatment strategies suggested an association between the portal vein tryptophan derivative level and the antibacterial defense of KCs in *Hamp1*^–/–^ mice. However, as the terminal product among the 3 sequential metabolites, IPA exhibits much higher physiological concentrations in portal blood compared with its intermediate precursor IA.

Taken together, untargeted metabolomics revealed that hepcidin deficiency altered portal blood metabolite profiles in mice, and in combination with further screening for specific tryptophan metabolites with gut microbiota depletion and fecal microbiota transplantation strategies, we found that the gut microbiota–derived tryptophan metabolite IPA might be a key gut–liver axis signaling molecule mediating the differences in the antibacterial defense of KCs between *Hamp1*^–/–^ and WT mice during bloodstream bacterial infection.

### IPA supplementation restores hepatic immune defense against bloodstream bacterial infection in Hamp1^–/–^ mice.

IPA, a tryptophan derivative produced exclusively in microorganisms, has been recently reported to play important bioactive roles in the progression of various diseases (38–40). We next sought to investigate whether IPA has a beneficial effect on hepatic immune defense against bloodstream bacterial infection in *Hamp1*^–/–^ mice. We first investigated whether IPA had any effect on the morphology of KCs from *Hamp1*^–/–^ mice ex vivo. Compared with PBS-treated KCs, IPA-stimulated KCs presented obvious morphological changes (Supplemental Figure 11, A and B), with significant increases in the volume (Supplemental Figure 11C) and surface area (Supplemental Figure 11D) of the cells. We further used bone marrow–derived macrophages (BMDMs) isolated from *Hamp1*^–/–^ mice to examine the effect of IPA on their phagocytic activity. IPA-pretreated BMDMs from *Hamp1*^–/–^ mice exhibited significantly enhanced phagocytosis of *E*. *coli*–GFP compared with the PBS-treated group (Supplemental Figure 11, E and F). Next, *Hamp1*^–/–^ mice were gavaged with IPA or sterile PBS for 7 days and thereafter subjected to bloodstream *E*. *coli* infection. Exogenous IPA supplementation significantly increased portal blood IPA concentrations in *Hamp1*^–/–^ mice (Figure 5F). Encouragingly, IPA treatment increased the bacterial capture capacity of KCs in *Hamp1*^–/–^ mice (Figure 5, G and H, and Supplemental Video 8). The improved hepatic antibacterial defense was further evidenced by increased sequestration of bacteria by the liver and a decreased circulating bacterial load at 2 hours after *E*. *coli* infection (Supplemental Figure 12, A and B), resulting in a decreased systemic bacterial burden at 24 hours after *E*. *coli* infection (Supplemental Figure 12, C and D). Moreover, we validated the effect of IPA on hepatic antibacterial defense in antibiotic-pretreated *Hamp1*^–/–^ and WT mice. Under conditions of gut microbiota depletion, IPA supplementation significantly improved hepatic bacterial sequestration and reduced circulating bacterial load both in antibiotic-pretreated *Hamp1*^–/–^ and WT mice at 2 hours after *E*. *coli* infection (Supplemental Figure 13, A and B). At 24 hours after *E*. *coli* infection, IPA supplementation decreased systemic bacterial burden in antibiotic-pretreated *Hamp1*^–/–^ and WT mice (Supplemental Figure 13, C and D).

We further assessed the morphological changes in KCs in *Hamp1*^–/–^ mice after IPA supplementation (Figure 5, I and J). As expected, IPA supplementation significantly increased the volume and surface area of KCs in *Hamp1*^–/–^ mice (Figure 5, K and L). These findings further support the hypothesis that IPA is a critical portal blood metabolite that modulates the hepatic immune defense against bloodstream bacterial infection in *Hamp1*^–/–^ mice.

### Colonization by IPA-producing L. intestinalis restores hepatic immune defense against bloodstream bacterial infection in Hamp1^–/–^ mice.

Changes in the profiles of gut microbiota–derived metabolites are closely associated with characteristic alterations in gut microecology. Thus, we next investigated whether there were any specific changes in gut microbes between *Hamp1*^–/–^ mice and WT mice. We detected gut microbial diversity and composition in fecal samples via 16S rDNA sequencing analysis. There was no significant difference in the gut microbiota composition at the phylum level between the 2 groups (Supplemental Figure 14A). Hepcidin deficiency did not significantly alter the microbiome biodiversity in the mouse intestine (Supplemental Figure 14, B and C). However, linear discriminant analysis effect size revealed that certain probiotics, such as *Lactobacillus*, *Akkermansia*, *Parabacteroides*, *Eubacterium*, and *Bacteroides*, were enriched in the intestines of WT mice but were scarce in the intestines of *Hamp1*^–/–^ mice (Supplemental Figure 14D). Moreover, a similar trend was found among the more abundant genera (top 20, accounting for 81.15% of the gut microbial composition), in which a substantial number of probiotics, including *Lactobacillus*, *Akkermansia*, *Alistipes*, and *Eubacterium*, were consistently reduced in abundance in the intestines of *Hamp1*^–/–^ mice (Supplemental Figure 14E).

**Figure 6:**
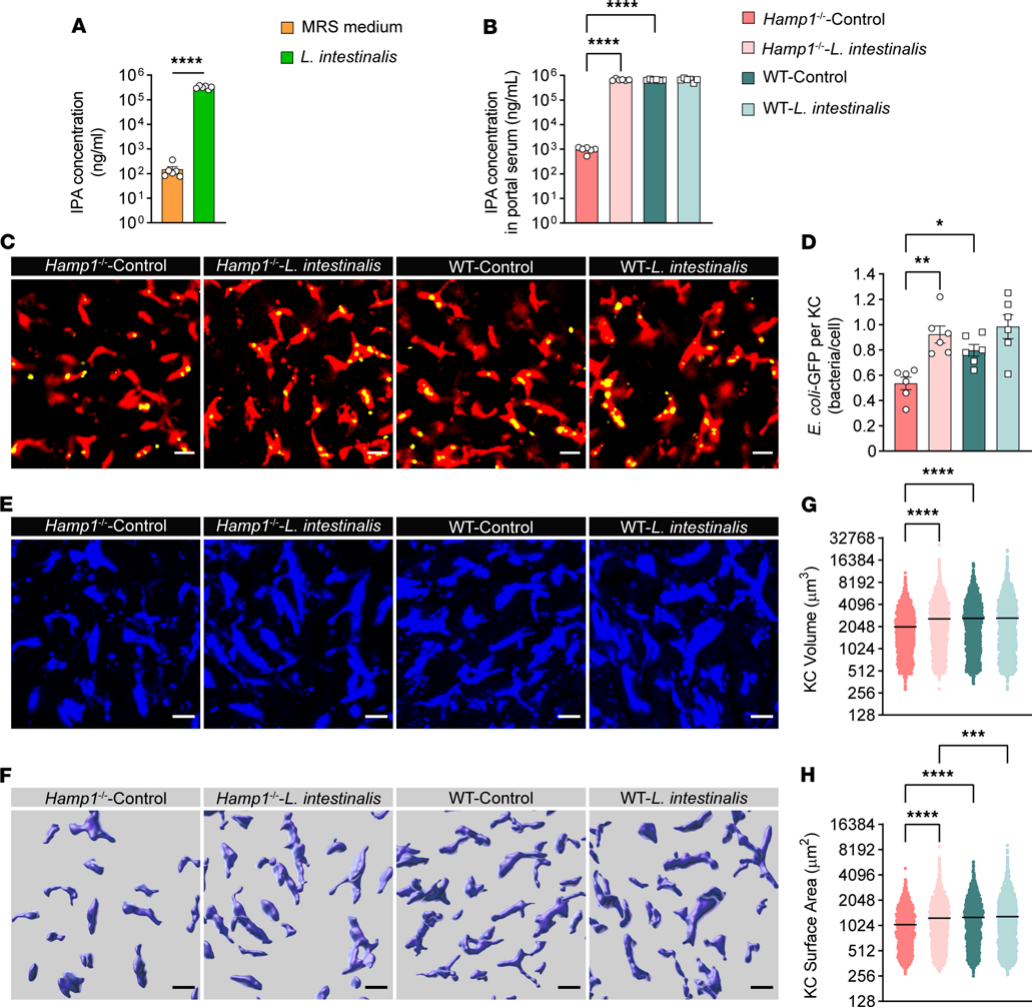
Colonization by IPA-producing *L.* *intestinalis* restores hepatic immune defense against bloodstream bacterial infection in *Hamp1*^–/–^ mice. (**A**) IPA concentration in the culture supernatant of *L*. *intestinalis* and Man, Rogosa, and Sharpe (MRS) medium. *n* = 6 per group; data are presented as mean ± SEM. (**B**) Portal blood level of IPA in mice pretreated with *L*. *intestinalis* or sterile PBS (control). *n* = 6 per group; data are presented as mean ± SEM. (**C**) Representative intravital microscopy (IVM) images showing KCs (red) capturing circulating *E*. *coli*–GFP (yellow) mice pretreated with *L*. *intestinalis* or sterile PBS (control). Scale bars: 20 μm. (**D**) Number of *E*. *coli*–GFP captured per KC. *n* = 6 per group; data are presented as mean ± SEM. (**E**–**H**) IVM image (**E**) combined with 3-dimensional reconstruction (**F**) to analyze KC volume (**G**) and surface area (**H**) in *Hamp1*^–/–^ and WT mice pretreated with *L*. *intestinalis* or sterile PBS (Control). Scale bars: 20 μm. *n* = 6 per group; data are presented as mean ± SEM. *P < 0.05, **P < 0.01, ***P < 0.001, ****P < 0.0001, by 2-tailed Student’s *t* test (**A**) or 1-way ANOVA followed by Šidák’s multiple-comparison test (**B**–**H**). Data presented are from 6 independent experiments (**A**–**H**). Each symbol represents an individual mouse (**B**–**D**). Symbols represent individual KCs from 6 mice with 5 fields of view per mouse (**G** and **H**).

Since members of the genus *Lactobacillus* have been reported to produce IPA, we further compared the abundance of *Lactobacillus* species between *Hamp1*^–/–^ mice and WT mice. Interestingly, among the 8 *Lactobacillus* species whose abundance was reduced in *Hamp1*^–/–^ mice, *L*. *intestinalis*, a functional probiotic of the genus *Lactobacillus*, was highly different in abundance between *Hamp1*^–/–^ mice and WT mice (Supplemental Figure 14F). After *L*. *intestinalis* was cultured overnight at 37°C under aerobic conditions with Man, Rogosa, and Sharpe medium, substantial increases in ILA, IA, and IPA concentrations were detected in the supernatant, with IPA showing the most pronounced elevation (Figure 6A, and Supplemental Figure 15, A and B). More importantly, after intestinal *L*. *intestinalis* colonization, the portal vein level of IPA in *Hamp1*^–/–^ mice was significantly elevated (Figure 6B). When the mice were further challenged with BSI with *E*. *coli*, intravital confocal microscopy analysis revealed that *L*. *intestinalis* colonization significantly promoted the bacterial capture ability of KCs in *Hamp1*^–/–^ mice but did not further increase this ability in WT mice (Figure 6, C and D, and Supplemental Video 9). Notably, *L*. *intestinalis* colonization improved the morphology of KCs in *Hamp1*^–/–^ mice (Figure 6, E and F). The KC volume and surface area in *Hamp1*^–/–^ mice colonized with *L*. *intestinalis* were markedly increased, whereas the KC morphology of WT mice was not significantly affected (Figure 6, G and H). We also observed that after *L*. *intestinalis* colonization, the volume of KCs in *Hamp1*^–/–^ mice was comparable with that in WT mice (Figure 6G), whereas the surface area of KCs in WT mice was still relatively larger than that in *Hamp1*^–/–^ mice (Figure 6H), which once again illustrates the much greater role of cell volume in controlling phagocytic efficiency.

Moreover, bacterial load assays performed at 2 and 24 hours after *E*. *coli* infection demonstrated that hepatic antibacterial defense was improved in *Hamp1*^–/–^ mice after *L*. *intestinalis* colonization (Supplemental Figure 16, A–D).

Taken together, these findings suggested that hepcidin deficiency disrupted the homeostasis of the gut microbiota, especially IPA-producing probiotics such as *L*. *intestinalis*, in mice. *L*. *intestinalis* colonization can enhance the antibacterial defense of KCs in *Hamp1*^–/–^ mice during bloodstream bacterial infection.

### Dietary iron restriction improves KC bacterial clearance and liver immune defense in Hamp1^–/–^ mice.

Hepcidin is a master hormonal regulator of systemic iron metabolism, and hepcidin deficiency can lead to severe iron overload in mice (41). To assess the potential impact of disturbed systemic iron on *Lactobacillus* abundance and IPA production, we employed WT mice fed a standard diet, a low-iron diet, or a high-iron diet for 4 weeks, followed by 16S rDNA sequencing analysis and quantitative IPA concentration assays. The WT mice fed a high-iron diet showed reduced abundance of *Lactobacillus* and decreased portal vein level of IPA compared with those fed a standard or low-iron diet (Supplemental Figure 17, A and B).

**Figure 7:**
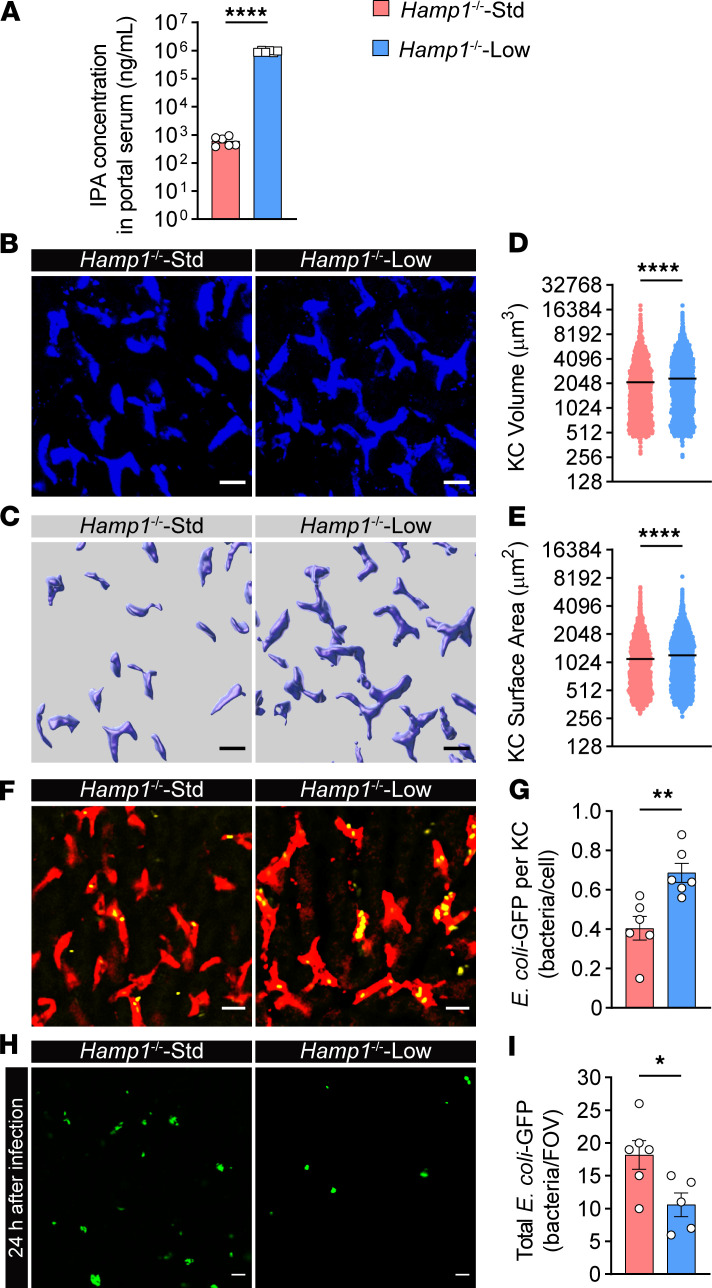
Dietary iron restriction improves KC bacterial clearance and liver immune defense in *Hamp1*^–/–^ mice. (**A**) Portal blood level of IPA in *Hamp1*^–/–^-Std (*Hamp1^–/–^* mice fed a standard diet) and *Hamp1*^–/–^-Low (*Hamp1^–/–^* mice fed a low-iron diet) mice. *n* = 6 per group; data are presented as mean ± SEM. (**B**–**E**) Intravital microscopy (IVM) image (**B**) combined with 3-dimensional reconstruction (**C**) to analyze KC volume (**D**) and surface area (**E**) in *Hamp1*^–/–^-Std and *Hamp1*^–/–^-Low mice. Scale bars: 20 μm. *n* = 6 per group; data are presented as mean ± SEM. (**F**) Representative IVM images showing KCs (red) capturing circulating *E*. *coli*–GFP (yellow) in *Hamp1*^–/–^-Std and *Hamp1*^–/–^-Low mice. Scale bars: 20 μm. (**G**) Number of *E*. *coli*–GFP captured per KC. *n* = 6 per group; data are presented as mean ± SEM. (**H**) Representative IVM images of the liver microcirculation at 24 h after *E*. *coli*–GFP infection. Scale bars: 20 μm. (**I**) Quantitative analysis of residual *E*. *coli*–GFP in the liver microcirculation (per field of view) at 24 h after *E*. *coli*–GFP infection. *n* = 5–6 per group; data are presented as mean ± SEM. *P < 0.05, **P < 0.01, ****P < 0.0001, by 2-tailed Student’s *t* test. Data presented are from 2 independent experiments (**A**–**I**). Each symbol represents an individual mouse (**A** and **F**–**I**). Symbols represent individual KCs from 6 mice with 5 fields of view per mouse (**D** and **E**).

We next sought to explore whether iron deprivation could improve the homeostasis of the gut–liver axis in *Hamp1*^–/–^ mice. Therefore, *Hamp1*^–/–^ mice were fed a low-iron or standard diet for 4 weeks. We first examined iron parameters in *Hamp1*^–/–^ mice after the iron-restricted diet. Iron load in the liver of *Hamp1*^–/–^ mice was significantly alleviated (Supplemental Figure 18A), while the iron load in the serum and spleen was comparable with that in the control mice (Supplemental Figure 18, B and C), which was consistent with previous reports (15, 42). We then measured portal vein serum levels of ILA, IA, and IPA. As expected, the mice fed the iron-restricted diet presented significantly greater concentrations of IPA in the portal vein (Figure 7A and Supplemental Figure 19, A and B), indicating that the homeostasis of the gut microbiota was restored in these mice. Consistent with this finding, the morphology of KCs in the mice fed a low-iron diet was synchronously ameliorated (Figure 7, B and C), as reflected by the improved volume and surface area (Figure 7, D and E).

We next assessed whether the iron-restricted diet had a positive effect on impaired hepatic antibacterial defense in *Hamp1*^–/–^ mice. As shown by hepatic intravital imaging, KCs from mice fed a low-iron diet exhibited better bacterial capture ability than did those from mice fed a standard iron diet (Figure 7, F and G, and Supplemental Video 10). Accordingly, at 2 hours after *E*. *coli* infection, the mice fed a low-iron diet had more effective hepatic bacterial sequestration and much less bacteremia (Supplemental Figure 20, A and B). Moreover, at 24 hours after *E*. *coli* infection, *Hamp1*^–/–^ mice fed a low-iron diet presented fewer residual hepatic bacteria (Figure 7, H and I) and lower systemic bacterial retention (Supplemental Figure 20, C–E). Restriction of iron intake in *Hamp1*^–/–^ mice also significantly ameliorated histologic damage to vital organs at 24 hours after infection (Supplemental Figure 20, F–I).

Together, the findings further demonstrate that hepcidin, a master regulator of systemic iron homeostasis, is important for liver immune defense against bloodstream bacterial infection by modulating the morphology of KCs through gut microbiota–derived metabolites.

### Hepcidin levels in patients with bacteremia correlate with their clinical status.

**Figure 8:**
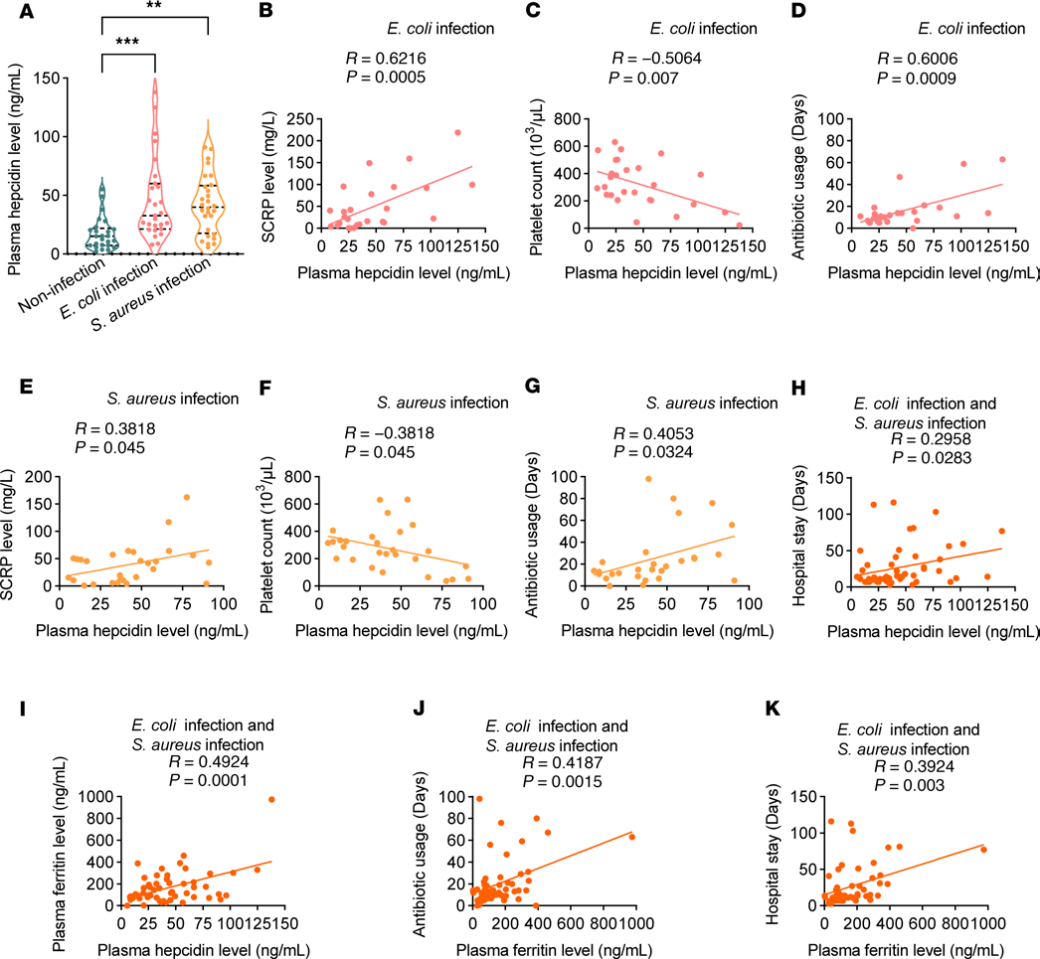
Hepcidin levels in patients with bacteremia correlate with their clinical status. (**A**) Plasma hepcidin levels in noninfectious controls (*n* = 25) and patients with *E*. *coli* bacteremia (*n* = 27) or with *S*. *aureus* bacteremia (*n* = 28). (**B**–**D**) Correlations of plasma hepcidin level with supersensitive C-reactive protein (SCRP) (**B**), platelet count (**C**), and days of antibiotic usage (**D**) in patients with *E*. *coli* bacteremia. (**E**–**G**) Correlations of plasma hepcidin level with SCRP (**E**), platelet count (**F**), and days of antibiotic usage (**G**) in patients with *S*. *aureus* bacteremia. (**H**) Correlation of plasma hepcidin level with hospital stay in all patients with bacteremia (*n* = 55). (**I**–**K**) Correlations of plasma ferritin level with plasma hepcidin level (**I**), days of antibiotic usage (**J**), and days of hospital stay (**K**) in all patients with bacteremia (*n* = 55). **P < 0.01, ***P < 0.001, by 1-way ANOVA followed by Tukey’s multiple-comparison test (**A**) or Pearson’s correlation analysis (**B**–**K**).

Hepcidin has been reported to be a sensitive acute-phase marker in critically ill patients (43). Hepcidin may allow early prediction and further facilitate early intervention as well as prognostic assessment of critical illness, especially sepsis. To echo the role of hepcidin in our murine model of bloodstream bacterial infection, we next sought to investigate the hepcidin level in patients with bacteremia and its relationship with the clinical characteristics of these patients. We enrolled 27 patients with positive blood cultures for *E*. *coli*, 28 patients with positive blood cultures for *S*. *aureus*, and 25 noninfected controls. The demographic and clinical characteristics of the cohort are listed in Supplemental Table 1. The plasma level of hepcidin was significantly greater in both groups of infected patients than in the controls (Figure 8A). In patients whose blood culture was positive for *E*. *coli*, the plasma hepcidin level was positively correlated with the supersensitive C-reactive protein level (Figure 8B) but negatively correlated with the platelet count (Figure 8C). Moreover, the plasma hepcidin level was positively correlated with the number of days of antibiotic usage (Figure 8D). Interestingly, similar findings were also discovered in patients whose blood cultures were positive for *S*. *aureus* (Figure 8, E–G). More importantly, when these 2 groups of patients were combined for analysis, the plasma hepcidin level was significantly positively correlated with the days of hospitalization (Figure 8H).

Hepcidin level is closely related to the systematic iron status. We further examined the major plasma iron parameters (iron, ferritin, and transferrin) in patients with bacteremia. We found that ferritin level showed a significant correlation with hepcidin concentrations in all patients with bacteremia, whereas circulating iron level and transferrin level did not (Figure 8I and Supplemental Figure 21, A and B). Furthermore, ferritin level positively correlated with the number of days of antibiotic usage and with the days of hospitalization in all patients with bacteremia (Figure 8, J and K).

Thus, the correlation between the plasma hepcidin and ferritin level and the clinical characteristics of patients with bacteremia further emphasized the critical role of hepcidin during bloodstream bacterial infection.

## Discussion

In the present study, we found that hepcidin and its orchestrated systemic iron sustained KC immune defense against bloodstream bacterial infections by modulating the gut commensal bacteria–derived tryptophan derivative IPA. Hepcidin deficiency compromised the hepatic defense against invading bacteria and exacerbated systemic bacterial dissemination. This deleterious effect was attributed to the morphological changes in KCs in *Hamp1*^–/–^ mice, which were caused by the reduced abundance of *L*. *intestinalis* in the gut microbiota and a concomitant reduction in its metabolite IPA in the portal vein. Moreover, exogenous IPA supplementation and commensal *L*. *intestinalis* colonization restored hepatic immune defense against bloodstream bacterial infections in *Hamp1*^–/–^ mice. In addition, the correlation between hepcidin levels and the clinical status of patients with bacteremia further highlights the importance of hepcidin in the host defense against bloodstream bacterial infections. Together, the results of the present study reveal a previously unappreciated role of hepcidin in KC immune defense against bloodstream bacterial infection through the regulation of gut microbial metabolite-mediated gut–liver communication.

The liver contains the largest pool of tissue-resident macrophages with a high capacity for repair and regeneration, properties that give it an unparalleled advantage in host defense against bacterial pathogen invasion. The ability of KCs to capture and kill bacterial pathogens is well documented (7, 44). We have also observed that KC depletion using clodronate liposomes diminished hepatic sequestration of circulating bacteria. However, although this has been the method of choice to deplete macrophages, clodronate liposomes have impacts on other cell types, including monocytes, dendritic cells, and neutrophils (45–47). Further study using other methods such as genetically modifying mice to selectively deplete KCs will elucidate their role in hepatic immune defense (48, 49). Key molecules/signaling pathways required for the homeostasis of KCs or their prevention of bacterial infections have been demonstrated (10, 27, 50). However, factors that regulate KC function in the eradication of invading bacteria remain largely unexplored. Our results demonstrated that hepcidin, the master regulator of systemic iron metabolism, prevents systemic bacterial dissemination (in both gram-positive and -negative bacterial infection models) by modulating the antibacterial defense of KCs. Notably, this effect is dependent on the regulatory role of hepcidin in linking the long-distance communication of gut commensal bacteria with KCs.

Mature KCs present different functional phenotypes and subpopulations (22, 23). The expansion and differentiation of KCs early in life are synchronized with the time of microbial colonization (24, 51). In the present study, we did not observe differences in the distributions of KC subpopulations between *Hamp1*^–/–^ and WT mice. However, KCs from *Hamp1*^–/–^ mice presented morphological defects and concomitant functional abnormalities in capturing circulating bacteria but were not deficient in the ability to kill intracellular bacteria, suggesting that hepcidin deficiency impaired circulating bacterial clearance by KCs primarily through altering KC morphology and then phagocytic efficiency but did not affect KC polarization or intracellular bacterial killing. Furthermore, RNA sequencing analysis with freshly isolated KCs revealed that hepcidin deficiency led to differentially expressed genes highly involved in phagocytosis-associated biological processes, further supporting the defective function of KCs against invading bacteria in *Hamp1*^–/–^ mice.

Previous studies have shown that a larger cellular volume facilitates the local expansion and contraction of phagocytosis pseudopods (33, 34), and for KCs, their morphology can be remotely modulated by the gut microbiota (26). Interestingly, our combined strategies of gut microbiota depletion and fecal microbiota transplantation not only demonstrated that the cellular volume, rather than the surface area, is decisive for phagocytic efficiency in *Hamp1*^–/–^ mice but also suggested that the gut microbiota engage in the long-lasting regulation of KC morphology/function, possibly through intrinsically derived signals.

We further explored the key signals downstream of hepcidin-mediated crosstalk between KCs and gut microbes. Anatomically, the liver is the first organ to collect venous return from the gut, and more than 70% of the blood enters the liver through the portal vein, which carries gut bacteria and microbial molecules from the gastrointestinal tract (3). Although largely unexplored, recent evidence has indicated that gut–liver crosstalk regulates the immune zonation and function of KCs. Persistent MYD88 (myeloid differentiation primary response protein 88)–dependent signaling induced by commensal bacteria leads to the asymmetric localization of KCs to periportal regions (8), and commensal bacteria–derived d-lactate reportedly promotes KC-dominated intravascular host defense against bacterial infection (26). Using untargeted metabolomics with further quantitative assays across different treatment strategies, we identified IPA, a gut microbiota–derived tryptophan derivative with significantly different concentrations in the portal blood between *Hamp1*^–/–^ and WT mice, as the key metabolite mediating hepatic antibacterial immune defense. Moreover, exogenous IPA supplementation significantly enhanced the bacterial capture capacity of KCs in *Hamp1*^–/–^ mice. Although the anti-inflammatory activity of IPA has recently been reported to exert organ-protective effects in sepsis (52–54), there are still relatively few studies regarding the effects of IPA on morphological properties of phagocytes, as well as bacterial uptake and killing of phagocytes. Therefore, the underlying mechanisms need further study.

We then continued to explore the differences in the gut microbial composition between *Hamp1*^–/–^ and WT mice and found that *L*. *intestinalis*, a member of the genus *Lactobacillus* with a reduced abundance in *Hamp1*^–/–^ mice, had a pronounced ability to produce IPA during culture. Furthermore, colonization with the commensal *L*. *intestinalis* reversed the functional abnormalities of KCs in *Hamp1*^–/–^ mice, and this ameliorative effect may be attributed to the improvement in KC morphology caused by IPA. These findings not only demonstrated a previously unknown long-lasting effect of hepcidin on host antibacterial immunity in addition to the iron deprivation of pathogenic microorganisms by inducing ferroportin degradation to induce acute-phase hypoferremia but also provided additional insights into the role of gut–liver long-distance communication in modulating hepatic antibacterial defense.

Previous studies reported that significantly higher hepcidin levels were observed in critically ill patients, with the highest levels in septic patients (55). Here, we found that plasma hepcidin levels were significantly greater in blood culture–positive patients than in noninfected controls. Moreover, hepcidin levels were positively correlated with days of in-hospital antibiotic use and length of hospitalization. The responsive increase in hepcidin during bacteremia indicates a stronger host response to invading bacteria. Notably, admission hepcidin levels have been reported to have high specificity with superior predictive value for long-term outcomes in patients with infections. Compared with those with low hepcidin levels, individuals with high hepcidin levels at admission had improved long-term survival (56, 57). Combined with the findings from the animal study, it will be valuable to follow up with the patients in the current study to illustrate the long-term significance of the responsive elevation of hepcidin in the early stages after bacteremia. Also, the findings in animal experiments will be further validated and extended in future clinical prospective studies that include portal vein IPA levels, bacterial loads, and fecal *Lactobacillus* abundance in patients with bacteremia.

In summary, our findings reveal a previously unappreciated long-lasting mechanism of hepcidin involvement in host defense. Hepcidin deficiency affects KC-mediated hepatic defense against bloodstream bacterial infections through the commensal *L*. *intestinalis* and its tryptophan derivative IPA. More importantly, from a translational perspective, restoring the crosstalk between the gut microbiota and liver through probiotic *L*. *intestinalis* colonization, dietary IPA supplementation, or dietary iron manipulation may offer a promising strategy for enhancing host defense against bloodstream bacterial infections in those with low hepcidin levels and a high risk of bacterial infection.

## Methods

### Sex as a biological variant.

All animal studies included equal representations of male and female mice. Sample collection involved both male and female patients.

See Supplemental Methods for additional information.

### Study approval.

The human study was approved by the Ethics Committee of Children’s Hospital, Zhejiang University School of Medicine, and informed consent was obtained from all patients or their surrogates. All of the animal studies were approved by the Laboratory Animal Welfare and Ethics Committee of Zhejiang University, and the handling of the animals was conducted in accordance with the NIH guidelines for ethical animal treatment.

### Data availability.

The raw RNA sequencing data have been deposited in the National Center for Biotechnology Information Gene Expression Omnibus database under accession number GSE297752. Data supporting the findings of this study are included in the main article and Supporting Data Values file, which is available online as supplemental material.

## Author contributions

QC, QS, and XF conceived and supervised the study, reviewed and edited the article, and acquired funding. YP, LS, XL, YZ, and SL designed the methods. YP, LS, ZW, XW, and QL performed investigations. YP and QC wrote the original draft.

## Supplementary Material

Supplemental data

Supplemental video 1

Supplemental video 2

Supplemental video 3

Supplemental video 4

Supplemental video 5

Supplemental video 6

Supplemental video 7

Supplemental video 8

Supplemental video 9

Supplemental video 10

Supporting data values

## Version 1 07/03/2025

In-Press Preview

## Version 2 09/02/2025

Electronic publication
