# Supplementary material for: Hepcidin sustains Kupffer cell immune defense against bloodstream bacterial infection via gut-derived metabolites in mice
Source: J Clin Invest. 2025 Jul 3;135(17):e189607. doi: 10.1172/JCI189607 (PMC12404757; doi:10.1172/JCI189607)
Supplement: Supplemental data [file jci-135-189607-s096.pdf]

Supplemental Methods

Sex as a biological variant

All animal studies included equal representations of male and female mice. Both male and female patients were involved for sample collection.

Animals

Hepcidin-1 knockout (*Hamp1*$^{-/-}$) mice with a C57BL/6 genetic background were generously provided by Dr. Sophie Vaulont (Institut Cochin, Paris, France) and Dr. Tomas Ganz (UCLA, Los Angeles, California) (1, 2). The sex- and age-matched littermates were used as wild-type mice. The mice were maintained in a specific pathogen-free mouse facility with food and water ad libitum. All of the animal studies were approved by the Animal Care and Use Committee of Zhejiang University (Hangzhou, China), and the handling of the animals was conducted in accordance with the National Institutes of Health guidelines for ethical animal treatment.

Mouse models

The *E. coli* strain (American Type Culture Collection [ATCC], 25922), *E. coli*-GFP strain (ATCC, 25922GFP), *S. aureus* strain (ATCC, 4300) and *S. aureus*-GFP strain (RN4220::sfGFP) were used to induce mouse models with bloodstream bacterial infection. For intravital imaging of hepatic bacteria capture, the mice received a single intravenous (i.v.) injection of 5.5×10^7 colony-forming units (CFUs) of *E. coli*-GFP or *S. aureus*-GFP. For intravital imaging of residual hepatic bacteria, the mice received a

single i.v. injection of 2×10^7 CFU of *E. coli*-GFP or 4.5×10^7 CFU of *S. aureus*-GFP. A single i.v. injection of 4.5×10^7 CFU of *E. coli* or 2×10^7 CFU of *S. aureus* was used to assess the bacterial burden after infection. For other experiments, a single i.v. injection of 4.5×10^7 CFU of *E. coli* or 1.75×10^7 CFU of *S. aureus* was used to induce bloodstream bacterial infection.

Bacterial burden assay

Bacterial burdens in the peripheral blood, liver and lungs were assessed. Briefly, after blood was collected in EDTA-coated tubes, mice were subjected to cardiac perfusion with sterile phosphate-buffered saline (PBS), then the liver and lung tissues were harvested and weighed before homogenization in cold sterile PBS. Tissue homogenates and blood samples were serially diluted 10-fold and inoculated onto Luria–Bertani agar plates, which were incubated at 37 °C for 12 to 16 hours under aerobic conditions. The numbers of bacterial colonies were calculated as CFU per milliliter of blood or CFU per gram of tissue.

Histopathological analysis

Mouse liver and lung tissues were harvested and fixed in 4% paraformaldehyde (PFA) for 48 hours and then embedded in paraffin wax. The sections (4 μm) were subjected to hematoxylin and eosin (H&E) staining following standard procedures as previously described and imaged on an Olympus VS120 Virtual Slide Microscope (Olympus, Tokyo, Japan) (3). The results of the quantitative histopathological analysis of liver and

lung injury were evaluated as previously described (4, 5). The injury score of each section was calculated as the average of at least 5 random visual fields.

Intravital microscopy

KCs were stained *in vivo* with Brilliant Violet 421-conjugated anti-F4/80 antibody (BioLegend, 123132, 1 μg/mouse) via *i.v.* injection 10 min before surgery. The bacterial bioparticles (pHrodo Green *E. coli* bioparticles, 200 μg/mouse, Thermo Fisher, P35366) used for phagosome acidification and the reporter bacteria used to detect ROS production were *i.v.* administered at the time of imaging. The bacteria used to detect ROS production were generated as previously described (6). Briefly, freshly prepared *E. coli* bacteria (5×10^8 CFU) were suspended in 500 μL of saline buffer containing 100 mM bicarbonate (pH 8.3). Then, Alexa Fluor 555 NHS ester (20 μg/mL, Thermo Fisher, A37571) and OxyBURST green H2DCFDA (60 μg/mL, Thermo Fisher, D2935) were added, followed by 30 min of incubation under vigorous agitation at room temperature. The OxyBURST green H2DCFDA was further activated in 250 μL of 1.5 M hydroxylamine hydrochloride (Sigma, 159417) on ice for 30 min, after which the reporter bacteria were washed twice with PBS. The labeling efficiency of the reporter bacteria was confirmed via confocal microscopy.

The surgical procedure for liver microscopy was performed as previously described (6). Briefly, after a midline incision in the abdomen was made from the pubis to the xiphoid process, the skin and abdominal musculature were removed to expose the liver. The xiphoid process was fixed with sutures tied in a knot to facilitate access to release the

falciform ligament. Each mouse was placed in the right lateral position, and the right lobe of the liver was gently exteriorized onto a coverslip. The lobe was gently covered by a small piece of wipe moistened with warm 1×PBS to eliminate breathing-related liver movement and achieve a flat area. Then, the liver was imaged on a Nikon A1R confocal microscope (Nikon, Tokyo, Japan) with a 20 × objective lens.

Intravital imaging analysis

NIS-Elements AR 4.51.00 (64-bit) software was used to acquire videos and images. Image analysis was performed using ImageJ (NIH) and Imaris (Olympus) software. Hepatic bacterial capture and clearance were quantified by the total number of individual bacteria per field of view (FOV) within 0.5–1.5 hours or at 24 hours after *E. coli*-GFP infection. Bacterial capture by KCs was quantified as the average number of bacterial particles captured by individual KCs per field of view (FOV). Phagosome acidification and reactive oxygen species (ROS) production in KCs were assessed by the mean fluorescence intensity (MFI) of pHrodo green *E. coli* bioparticles and OxyBURST green H2DCFDA phagocytosed by KCs per field of view (FOV). The number of KCs, cell volume and surface area were measured via three-dimensional reconstruction with Imaris software.

KC depletion *in vivo*

To deplete KCs *in vivo*, the mice received a single *i.v.* injection of 100 μL of liposome-encapsulated clodronate or control liposomes (FormuMax, F70101C-NC-10) 48 hours

prior to induction of the bloodstream bacterial infection model. Depletion efficiency was confirmed by flow cytometry.

Flow cytometry

Mouse hepatic nonparenchymal cells (NPCs) were isolated for KC phagocytosis analysis and for the phenotypic characterization of KC subsets via flow cytometry as previously described (7). KCs were gated on $CD45^+$ $F4/80^+$ $CD11b^{int}$ $MHCII^+$ $TIM4^+$ hepatic nonparenchymal cells, as shown in Supplemental Figure 5C. The efficiency of *E. coli*-GFP uptake by KCs was quantified as the MFI of *E. coli*-GFP in KCs. The KC subsets were identified as $CD45^+$ $F4/80^+$ $CD11b^{int}$ $MHCII^+$ $TIM4^+$ $ESAM^-$ $CD206^-$ (KC1) and $CD45^+$ $F4/80^+$ $CD11b^{int}$ $MHCII^+$ $TIM4^+$ $ESAM^+$ $CD206^+$ (KC2) hepatic nonparenchymal cells (8), followed by dimensionality reduction with t-distributed stochastic neighbor embedding (tSNE) using FlowJo software and quantification of the proportion of total $CD45^+$ $F4/80^+$ $CD11b^{int}$ cells. Data acquisition was performed on a BD LSRFortessa flow cytometer (BD Biosciences), and data analysis was performed with FlowJo, version 10.6.2.

Immunofluorescence staining

Liver tissues were harvested and fixed in 4% paraformaldehyde (PFA) for 24 hours, followed by dehydration in 30% sucrose before being embedded in OCT compound (Sakura, 4583). Frozen sections (10 μm) of livers were cut on an NX50 cryostat (Thermo Fisher) and adhered to Citotest adhesion microscope slides. The sections were

permeabilized with 0.3% Triton X-100, blocked with 5% BSA in PBS, and then stained with an anti-F4/80 antibody (Thermo Fisher, MA5-16630) at 4 °C overnight. Following washing with PBST (PBS containing 0.05% Tween 20), the sections were stained with Alexa Fluor 647-conjugated donkey anti-rat IgG (Thermo Fisher, A48272TR) for 1 hour at room temperature. After being washed with PBST, the sections were stained with DAPI in antifade mounting medium (Beyotime, P0131). Images were captured on an Olympus FV3000 microscope (Olympus, Tokyo, Japan) with a 40 × UPLANSAPO objective lens.

RNA sequencing

Mouse hepatic non-parenchymal cells were isolated as previously described (7). For fluorescence-activated cell sorting (FACS), KCs were gated on live $CD45^+$ $F4/80^+$ $CD11b^{int}$ hepatic nonparenchymal cells on a Beckman MoFlo Astrios EQ (Beckman Coulter Life Sciences, Indianapolis, IN, USA). Samples were commissioned to LC-Bio Technology Co., Ltd. (Hangzhou, China) to perform switching mechanism at the 5′ end of the RNA transcript (SMART-seq). RNA was extracted and amplified using a SMART-Seq Stranded Kit (Takara Bio USA), and the experiments were performed according to the manufacturer’s standard protocols. Sequencing was conducted on the Illumina 6000 sequencing platform at LC-Biotechnology. Genes differential expression analysis was performed by DESeq2 software. The cutoff was set as p value < 0.05 and |log2FC| ≥1. Differentially expressed genes were then subjected to enrichment analysis of KEGG pathways. The raw data have been deposited in the NCBI Gene Expression

Omnibus (GEO) database with accession number GSE297752.

Antibiotic treatment

For the gut microbiota depletion experiments, the mice were gavaged with either an antibiotic cocktail (4Abx) consisting of vancomycin (0.5 mg/mL, MCE, HY-17362), ampicillin (1 mg/mL, Sigma, A5354), metronidazole (1 mg/mL, Sigma, M1547) or neomycin (0.5 mg/mL, Sigma, N6386) in 200 μL sterile water or 200 μL sterile water for 21 days as previously described (9), followed by a 5-day washout period during which the mice were given sterile water.

Fecal microbiota transplantation (FMT)

Healthy *Hamp1*$^{-/-}$ or WT mice were placed in a clean cage, and fecal pellets were collected in a cryotube. The pooled feces (approximately 1 gram) were then homogenized in 10 mL of PBS by vortexing followed by centrifugation at 4 °C for 30 s at 3,000 rpm. The supernatant was subsequently transferred to a new tube and centrifuged at 4 °C for 5 min at 12,000 rpm. Afterward, the pellet was resuspended in 2.5 mL of sterile PBS. After the recipient mice were pretreated with the antibiotic cocktail (4Abx) for 21 days, fecal microbiota transplantation was carried out daily by oral gavage with 500 μL of fecal microbiota for 10 days (10).

Metabolomic analysis

Untargeted metabolomics analysis was performed using Liquid Chromatography Mass

Spectrometry, LC-MS). The portal serum samples stored at -80°C were thawed on ice, and metabolites were extracted from 20 μL of each sample with 120 μL of precooled 50% methanol buffer. The mixture of metabolites was subsequently vortexed for 1 min, incubated for 10 min at room temperature, and stored at -20°C overnight. Samples were commissioned to LC-Bio Technology Co., Ltd. (Hangzhou, China). Briefly, the mixture was centrifuged at 4,000 × g for 20 min, and subsequently, the supernatant was transferred to 96-well plates. In addition, pooled quality control samples were also prepared by combining 10 μL of each extraction mixture. The Chromatographic separation was performed using an UltiMate 3000 UPLC System (Thermo Fisher Scientific, Bremen, Germany). An ACQUITY UPLC T3 column (100 mm × 2.1 mm, 1.8 μm, Waters, Milford, USA) was used for reversed-phase separation. A high-resolution tandem mass spectrometer TripleTOF 6600 (SCIEX, Framingham, MA, USA) was used to detect metabolites eluted form the column. The raw data files were converted into mzXML format and then processed using the XCMS, CAMERA, and metaX toolboxes included in R software. Each ion was identified by the combined information of retention time and m/z data. The intensity of each peak was recorded, and a three-dimensional matrix containing arbitrarily assigned peak indices (retention time-m/z), sample names (observations) and ion intensity information (variables) was generated. Using the open-access databases KEGG and the human metabolome database, metabolites were annotated by matching the exact molecular mass data (m/z) with those in the databases within a threshold of 10 ppm. The peak intensity data were further preprocessed via metaX. Features that were detected in < 50% of the QC

samples or 80% of the test samples were removed. PCA was performed to detect outliers and batch effects via the preprocessed dataset, and metabolite set enrichment analysis was performed for significantly differentially expressed metabolites with absolute values of fold change ≥ 1.2, p value < 0.05 and VIP ≥ 1.

ILA, IA and IPA detection were commissioned to Majorbio BioPharm Technology Co., Ltd. (Shanghai, China) using targeted LC-MS/MS analysis as previously described (11). Briefly, for portal serum samples, pre-cooled methanol was added in 5-10 times of volume, and the mixture was vortexed, shaken, and incubated for 1 hour at -20°C. After centrifuging for 10 min at 14,000 × g, the supernatant was collected and centrifuged again. The supernatant was then filtered for further assay. For culture supernatants, after centrifuging for 10 min at 14,000 × g to remove the organisms and impurities, the supernatant was acidified to pH = 2.5 using hydrochloric acid and extracted twice with double volume of ethyl acetate. Then, the samples were air-dried and redissolved in one-tenth volume of methanol, and filtered for further assay. Metabolites were detected on the 5500 QTRAP triple quadrupole mass spectrometer (SCIEX, Framingham, MA, USA). The analytical conditions were as follows: buffer A was ddH_2O plus 0.1% formic acid and buffer B was acetonitrile plus 0.1 % formic acid. The gradient conditions were as follows: 0-1 min, 5 % buffer B; 1-7 min, gradient to 95% buffer B; 7-10 min, 95% buffer B; 10-13 min, gradient to 5 % buffer B; 13-14 min, 5 % buffer B. The flow rate was 0.4 mL/min.

IPA treatment

For IPA intervention, a storage solution of purified IPA (Aladdin, I103959) was diluted in sterile PBS (final concentration 5 mg/mL), and the mice were gavaged with IPA (50 mg/kg) daily for 7 days (12). The control group was gavaged with an equal volume of sterile PBS.

Cell culture and treatment

Hepatic nonparenchymal cells (NPCs) from *Hamp1*$^{-/-}$ mice were isolated as previously described (7). For fluorescence-activated cell sorting (FACS), KCs were gated on live $CD45^{+}$ $F4/80^{+}$ $CD11b^{int}$ hepatic nonparenchymal cells on a Beckman MoFlo Astrios EQ (Beckman Coulter Life Sciences, Indianapolis, IN, USA). The sorted KCs were washed twice with sterile PBS and then resuspended in Dulbecco's modified Eagle's medium (DMEM) supplemented with 10% FBS and 1% penicillin–streptomycin and were maintained in a humidified incubator (Thermo Fisher Scientific, Rockford, IL) with 5% CO_2 at 37°C. To assess the effect of IPA on KC morphology, KCs were treated with IPA (100 μM, Aladdin, I103959) or sterile PBS during culture. Twenty-four hours after IPA treatment, the KCs were stained with Brilliant Violet 421-conjugated anti-F4/80 antibody (BioLegend, 123132) and imaged on an Olympus FV3000 microscope (Olympus, Tokyo, Japan) with a 20 × objective lens.

Bone marrow-derived macrophages (BMDMs) from *Hamp1*$^{-/-}$ mice were isolated as previously described (13). After isolation, the bone marrow cells were resuspended in DMEM supplemented with 10% fetal bovine serum and were maintained in a humidified incubator (Thermo Fisher Scientific, Rockford, IL) with 5% CO_2 at 37°C.

Twenty-four hours later, the nonadherent cells were collected and incubated for 7 days with medium exchange every 3 days. To assess the effect of IPA on phagocytic activity, BMDMs were treated with IPA (100 μM, Aladdin, I103959) or sterile PBS during culture. Before experiments, the BMDMs were stained with Brilliant Violet 421-conjugated anti-F4/80 antibody (BioLegend, 123132) and challenged with heat-inactivated *E. coli*-GFP at a multiplicity of infection of 25 for 30 min, followed by termination of phagocytosis with pre-cooled PBS at 4°C. Then the BMDMs were imaged on an Olympus FV3000 microscope (Olympus, Tokyo, Japan) with a 40 × objective lens.

16S rDNA sequencing

Healthy *Hamp1*$^{-/-}$ or WT mice or WT mice subjected to dietary iron manipulation were placed in separate clean cages, and 1-2 fecal pellets were collected in a cryotube. All collected samples were immediately frozen in liquid nitrogen and stored at -80°C for future analysis. For 16S rDNA gene sequencing, total DNA was isolated from mouse fecal samples via cetyltrimethyl ammonium bromide (CTAB), and its quality was examined using 1% agarose gel electrophoresis. The V3-V4 regions of the 16S rDNA genes were amplified using the primers 341F (5'-CCTACGGGNGGCWGCAG-3') and 805R (5'-GACTACHVGGGTATCTAATCC-3') and a thermocycler PCR system (LC-Bio Technology Co., Ltd., Hangzhou, China). The PCR products were confirmed via 2% agarose gel electrophoresis, purified with AMPure XT beads (Beckman Coulter Genomics, Danvers, MA, USA), and then quantified with a Qubit instrument

(Invitrogen, Carlsbad, CA, USA). The sequencing library was generated using an Agilent 2100 Bioanalyzer (Agilent, Palo Alto, CA, USA) and the Library Quantification Kit for Illumina (Kapa Biosciences, Woburn, MA, USA). The libraries were sequenced on the NovaSeq 6000 platform. Alpha diversity was conducted to assess the complexity of the species diversity of each sample using QIIME2 software. Beta diversity was also analyzed via PCoA with QIIME2 software to examine the diversity in samples between the groups. LEfSe analysis was employed to assess the differentially abundant taxa.

L. intestinalis culture and colonization

Single colonies of *L. intestinalis* (Deutsche Sammlung von Mikroorganismen und Zellkulturen [DSMZ], DSM 6629) were grown in de Man, Rogosa and Sharpe (MRS) medium in a shaking incubator at 37°C overnight under aerobic conditions. The medium was then spun down, and the bacterial pellets were resuspended in sterile PBS. The mice were gavaged with 10^8 CFU of fresh cultured *L. intestinalis* or sterile PBS daily for 7 days.

Dietary iron manipulation

The standard diet, low-iron diet and high-iron diet were obtained from Jiangsu Xietong Pharmaceutical Bioengineering Co., Ltd., China. The diet formulation was based on AIN-93G, and the dietary iron concentrations were 45 ppm (standard diet, XT93G), less than 5 ppm (low-iron diet, XTLFe01), and 8000 ppm (high-iron diet, XTMM) respectively. The mice were fed the diet ad libitum for 4 weeks, starting at 4 weeks of

age (14). After feeding, iron load in liver, spleen and serum was measured using a commercial non-heme iron assay kit (LEAGENE, TC1000) according to the manufacturer's instructions.

Human subjects

From March 2022 to April 2024, patients who were suspected of having bloodstream infections after admission to the Children's Hospital Zhejiang University School of Medicine were initially screened for eligibility. Patients who were diagnosed with cancer, human immunodeficiency virus infection, renal disease, or viral hepatitis or were unable to provide informed consent were excluded. Age- and sex-matched noninfectious patients were enrolled as controls. Clinical data, including laboratory routine examinations, microbial species, in-hospital antibiotic days, and length of ICU stay and hospital stay, were recorded. Blood samples were simultaneously collected for measurement of hepcidin levels when patients were advised to undergo microbiological inspection. Blood samples were centrifuged at 3000 rpm for 15 minutes to obtain plasma, which was stored at -80°C for further analysis. Plasma hepcidin-25 measurements were performed with an ELISA kit (DRG, EIA-5782) according to the manufacturer's instructions. Plasma iron concentration was measured using a commercial non-heme iron assay kit (LEAGENE, TC1000) according to the manufacturer's instructions. Plasma ferritin and transferrin levels were detected using commercial ELISA kits (Abcam, ab108837, ab187391). This study was approved by the local Ethics Committee, and informed consent was obtained from all patients or

their surrogates.

Statistics

All data are presented as the mean ± SEM or as the median with range where applicable and unless otherwise stated. Differences between two groups were analyzed by two-tailed Student's *t* test or the Mann–Whitney *U* test. Multigroup comparisons were assessed via one-way ANOVA followed by Šidák's multiple-comparison test or Tukey's multiple-comparison test, or via the Kruskal–Wallis followed by Dunn's multiple-comparison test where applicable. Correlations were determined via Pearson's correlation analysis. The survival curves were analyzed by the Kaplan–Meier log-rank test. The statistical analyses were performed using GraphPad Prism software 9.5 (GraphPad Software Inc.). A P value less than 0.05 was considered significant.

References

1. Viatte L, Lesbordes-Brion JC, Lou DQ, Bennoun M, Nicolas G, Kahn A, et al. Deregulation of proteins involved in iron metabolism in hepcidin-deficient mice. *Blood.* 2005;105(12):4861-4.
2. Ramos E, Ruchala P, Goodnough JB, Kautz L, Preza GC, Nemeth E, et al. Minihepcidins prevent iron overload in a hepcidin-deficient mouse model of severe hemochromatosis. *Blood.* 2012;120(18):3829-36.
3. Chen Q, Yang Y, Hou J, Shu Q, Yin Y, Fu W, et al. Increased gene copy number of DEFA1/DEFA3 worsens sepsis by inducing endothelial pyroptosis. *Proc Natl Acad Sci U S A.* 2019;116(8):3161-3170.
4. Ito S, Tanaka Y, Oshino R, Okado S, Hori M, and Isobe KI. GADD34 suppresses lipopolysaccharide-induced sepsis and tissue injury through the regulation of macrophage activation. *Cell Death Dis.* 2016;7(5):e2219.
5. Kawamura T, Huang CS, Peng X, Masutani K, Shigemura N, Billiar TR, et al. The effect of donor treatment with hydrogen on lung allograft function in rats. *Surgery.* 2011;150(2):240-9.
6. Surewaard BGJ, and Kubes P. Measurement of bacterial capture and phagosome maturation of Kupffer cells by intravital microscopy. *Methods.* 2017;128:12-19.
7. Andreata F, Blériot C, Di Lucia P, De Simone G, Fumagalli V, Ficht X, et al. Isolation of mouse Kupffer cells for phenotypic and functional studies. *STAR Protoc.* 2021;2(4):100831.
8. De Simone G, Andreata F, Bleriot C, Fumagalli V, Laura C, Garcia-Manteiga JM, et al. Identification of a Kupffer cell subset capable of reverting the T cell dysfunction induced by hepatocellular priming. *Immunity.* 2021;54(9):2089-2100.e8.
9. Schieber AM, Lee YM, Chang MW, Leblanc M, Collins B, Downes M, et al. Disease tolerance

mediated by microbiome E. coli involves inflammasome and IGF-1 signaling. *Science.* 2015;350(6260):558-63.

10. Serger E, Luengo-Gutierrez L, Chadwick JS, Kong G, Zhou L, Crawford G, et al. The gut metabolite indole-3 propionate promotes nerve regeneration and repair. *Nature.* 2022;607(7919):585-592.

11. Wikoff WR, Anfora AT, Liu J, Schultz PG, Lesley SA, Peters EC, et al. Metabolomics analysis reveals large effects of gut microflora on mammalian blood metabolites. *Proc Natl Acad Sci U S A.* 2009;106(10):3698-703.

12. Xue H, Chen X, Yu C, Deng Y, Zhang Y, Chen S, et al. Gut Microbially Produced Indole-3-Propionic Acid Inhibits Atherosclerosis by Promoting Reverse Cholesterol Transport and Its Deficiency Is Causally Related to Atherosclerotic Cardiovascular Disease. *Circ Res.* 2022;131(5):404-420.

13. Chen Q, Zhang K, Jin Y, Zhu T, Cheng B, Shu Q, et al. Triggering receptor expressed on myeloid cells-2 protects against polymicrobial sepsis by enhancing bacterial clearance. *Am J Respir Crit Care Med.* 2013;188(2):201-12.

14. Bessman NJ, Mathieu JRR, Renassia C, Zhou L, Fung TC, Fernandez KC, et al. Dendritic cell-derived hepcidin sequesters iron from the microbiota to promote mucosal healing. *Science.* 2020;368(6487):186-189.

Supplemental Figures

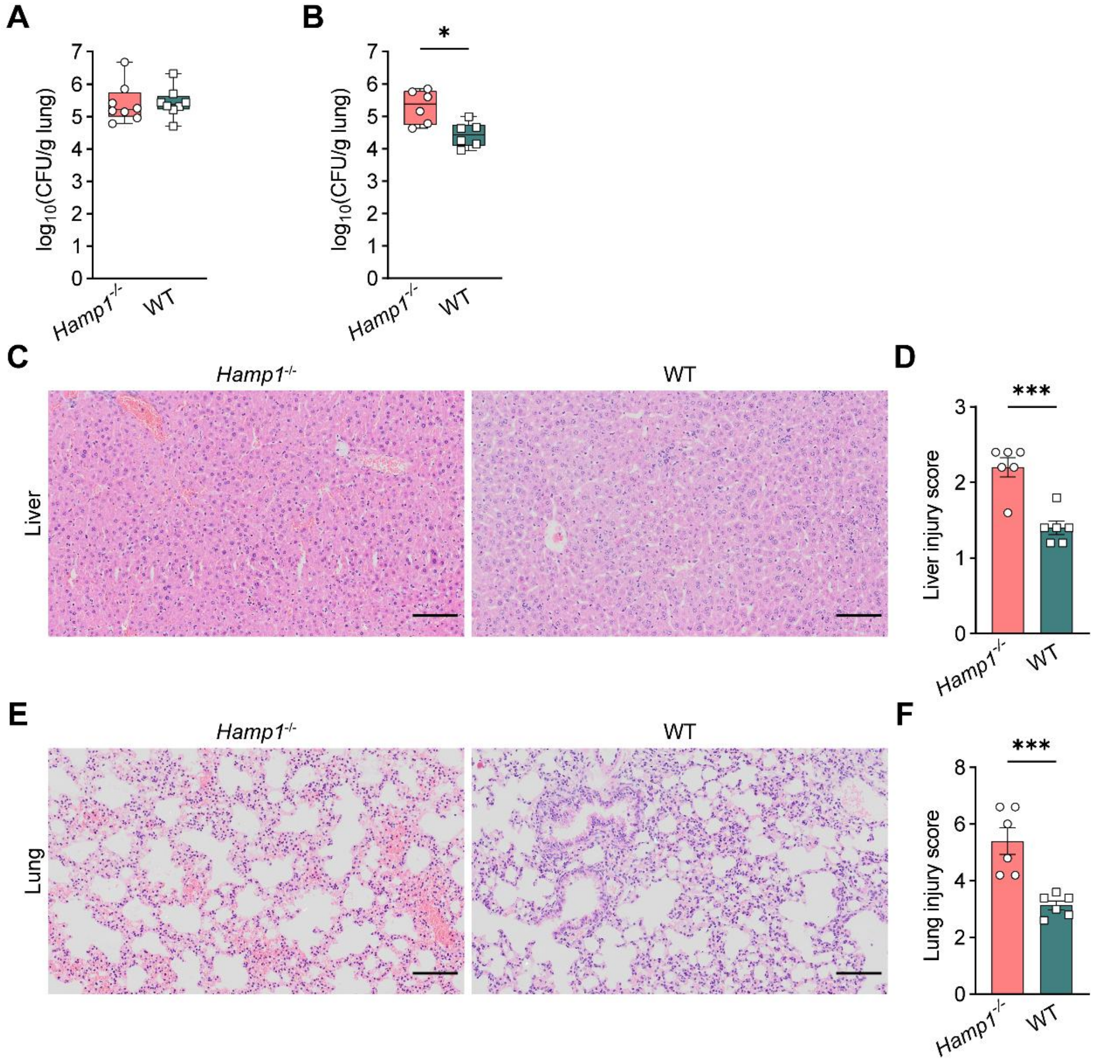

Fig. S1. Hepcidin deficiency exacerbates systemic dissemination of bacteria to vital organs and aggravates organ damage. (**A**) Bacterial load in the lungs at 2 h after *E. coli* infection in *Hamp1*$^{-/-}$ and WT mice. n = 8 per group, data are presented as median ± interquartile range. (**B**) Bacterial load in the lungs at 24 h after *E. coli* infection in *Hamp1*$^{-/-}$ and WT mice. n = 6 per group, data are presented as median ± interquartile range. (**C**) Representative images of livers with H&E staining at 24 h after *E. coli* infection in *Hamp1*$^{-/-}$ and WT mice. Scale bar, 50 µm. (**D**) Quantification of liver injury score. n = 6 per group, data are presented as mean ± SEM. (**E**) Representative images of lungs with H&E staining at 24 h after *E. coli* infection in *Hamp1*$^{-/-}$ and WT mice. Scale bar, 50 µm. (**F**) Quantification of lung injury score. n = 6 per group, data are presented as mean ± SEM. **p* < 0.05, ****p* < 0.001, by Mann–Whitney *U* test (A and B), two-tailed Student's *t* test (C-F). Data presented are from 8 (A-B) and 3 (C-F) independent experiments. Each symbol represents an individual mouse. *E. coli*, *Escherichia coli*; H&E, hematoxylin and eosin; CFU, colony forming units.

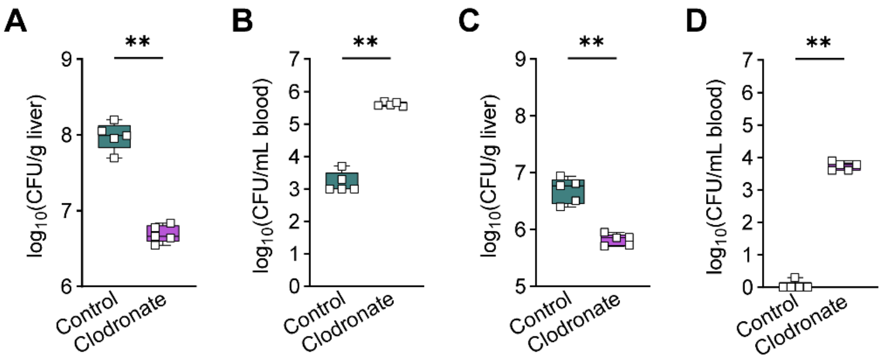

Fig. S2. KCs in hepatic defense against bloodstream bacterial infection. (**A** and **B**) Bacterial load in the livers (**A**) and peripheral blood (**B**) at 2 h after *E*. coli infection in WT mice pretreated with control liposomes (Control) or clodronate liposomes (Clodronate). n = 5 per group, data are presented as median ± interquartile range. (**C** and **D**) Bacterial load in the livers (**C**) and peripheral blood (**D**) at 24 h after *E*. coli infection in WT mice pretreated with control liposomes (Control) or clodronate liposomes (Clodronate). n = 5 per group, data are presented as median ± interquartile range. $^{**}p < 0.01$, by Mann–Whitney *U* test. Data presented are from 3 (A-B) and 2 (C-D) independent experiments. Each symbol represents an individual mouse. KC, Kupffer cell; *E. coli*, *Escherichia coli*; CFU, colony forming units.

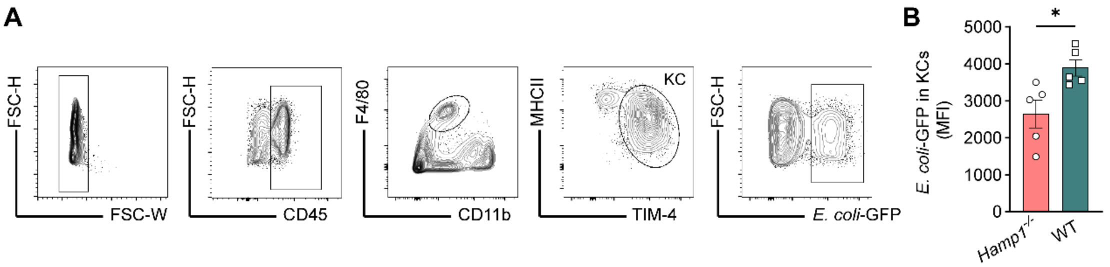

Fig. S3. KC bacteria capture assessed by flow cytometry. (**A**) Gating strategy for *E. coli*-GFP phagocytosed by KCs. (**B**) MFI of *E. coli*-GFP engulfed by KCs. n = 5 per group, data are presented as mean ± SEM. *$p < 0.05$, by two-tailed Student's *t* test. Data presented are from 4 independent experiments. Each symbol represents an individual mouse. KC, Kupffer cell; *E. coli*, *Escherichia coli*; GFP, green fluorescent protein; MFI, mean fluorescence intensity.

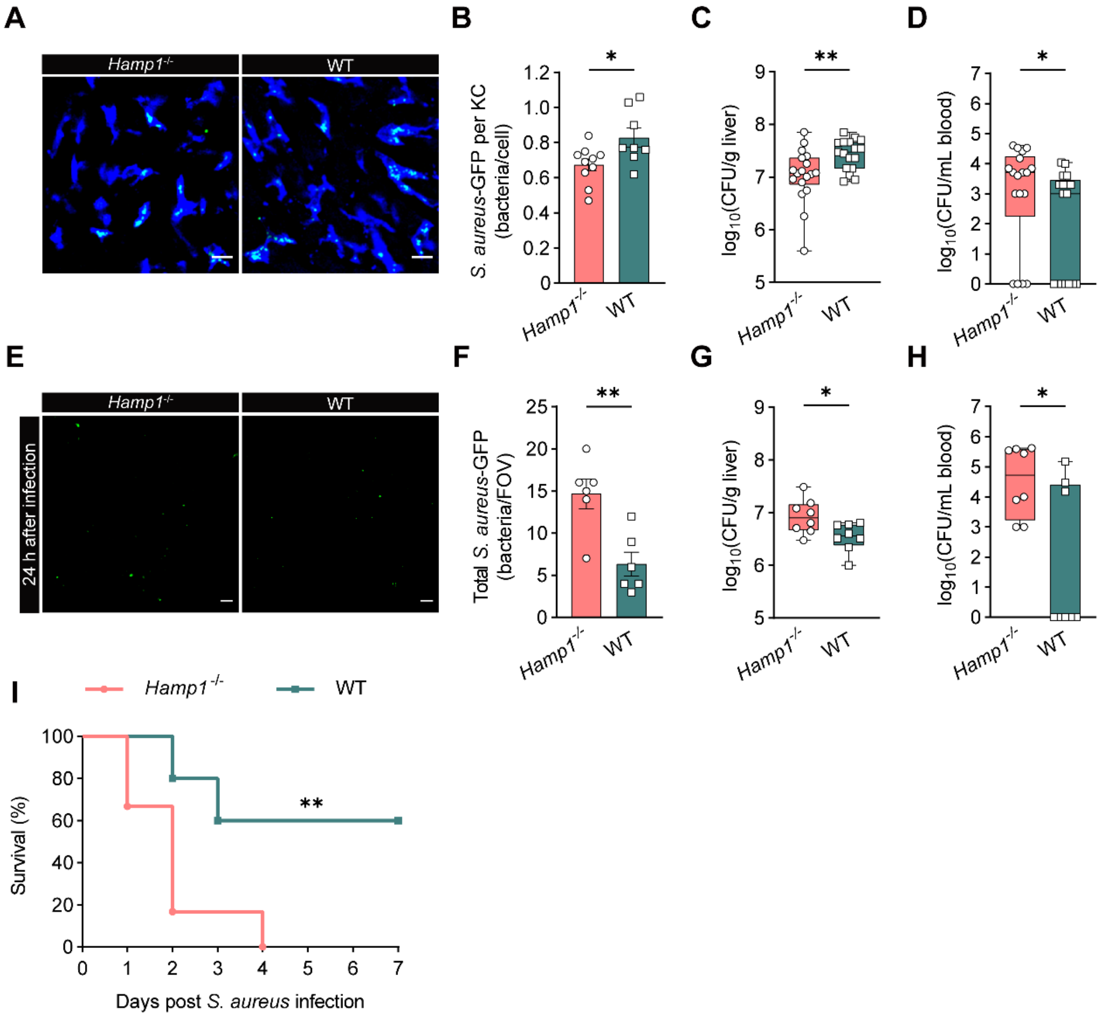

Fig. S4. Hepcidin deficiency impairs circulating *S. aureus* clearance by KCs. (**A**) Representative IVM images showing KCs (blue) capturing circulating *S. aureus*-GFP (green) in $Hamp1^{-/-}$ and WT mice. Scale bars, 20 μm. (**B**) Number of *S. aureus*-GFP captured per KC. n = 8-10 per group, data are presented as mean ± SEM. (**C** and **D**) Bacterial load in the livers (**C**) and peripheral blood (**D**) at 2 h after *S. aureus* infection in $Hamp1^{-/-}$ and WT mice. n = 17-18 per group, data are presented as median ± interquartile range. (**E**) Representative IVM images of the liver microcirculation at 24 h after *S. aureus* infection in $Hamp1^{-/-}$ and WT mice. Scale bars, 20 μm. (**F**) Residual *S. aureus*-GFP in the livers (per FOV) at 24 h after infection. n = 6 per group, data are presented as mean ± SEM. (**G** and **H**) Bacterial load in the livers (**G**) and peripheral blood (**H**) at 24 h after *S. aureus* infection in $Hamp1^{-/-}$ and WT mice. n = 8 per group, data are presented as median ± interquartile range. (**I**) Survival rate of $Hamp1^{-/-}$ and WT mice after *S. aureus* infection. n = 6-10 per group. *$p < 0.05$, ** $p < 0.01$, by two-tailed Student's *t* test (A-B and E-F), Mann–Whitney *U* test (C-D and G-H), and Kaplan–Meier log-rank test (I). Data presented are from 7 (A-B), 8 (C-D), 5 (G-H) and 2 (I) independent experiments. Each symbol represents an individual mouse. *S. aureus*, *Staphylococcus aureus*; KC, Kupffer cell; GFP, green fluorescent protein; CFU, colony forming units; FOV, field of view.

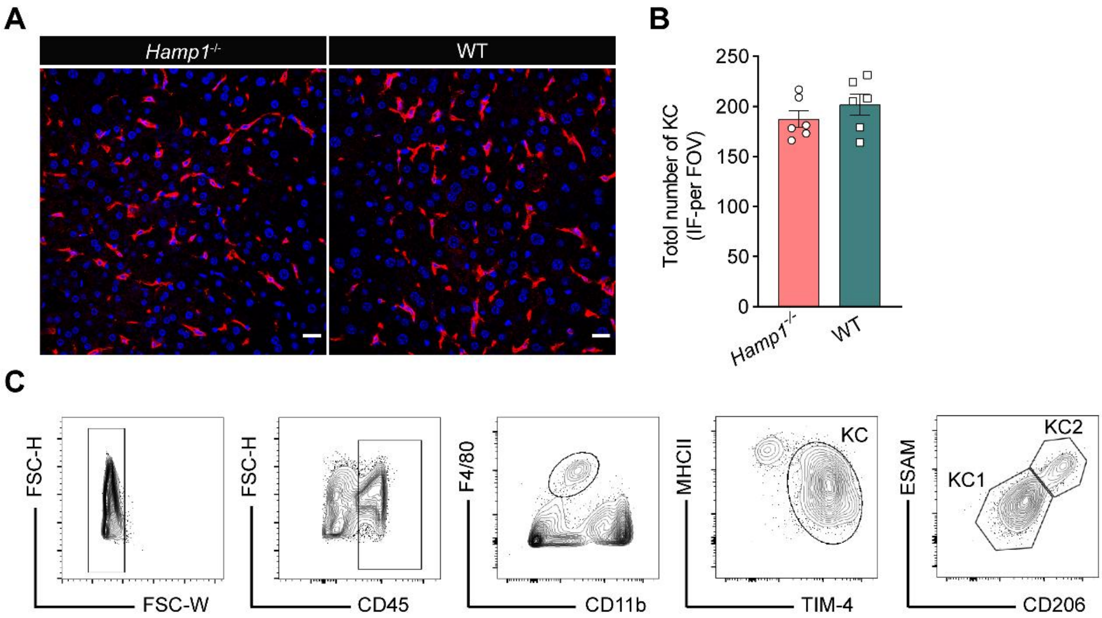

Fig. S5. Immunofluorescence quantification of KC number and flow cytometry gating strategy for KC subpopulations. (A) Representative immunofluorescence images of KCs in *Hamp1*$^{-/-}$ and WT mice (red, F4/80; blue, DAPI). Scale bars, 20 μm. (B) Total number of KCs (per FOV) in *Hamp1*$^{-/-}$ and WT mice. n = 6 per group, data are presented as mean ± SEM. (**C**) Gating strategy for KCs subpopulations. KC1 was identified as CD45^{+} F4/80^{+} CD11b^{int} MHC II^{+} TIM4^{+} ESAM^{-} CD206^{-} cells, and KC2 was identified as CD45^{+} F4/80^{+} CD11b^{int} MHC II^{+} TIM4^{+} ESAM^{+} CD206^{+} cells. By two-tailed Student's *t* test. Data presented are from 5 independent experiments. Each symbol represents an individual mouse. KC, Kupffer cell; IF, immunofluorescence; FOV, field of view.

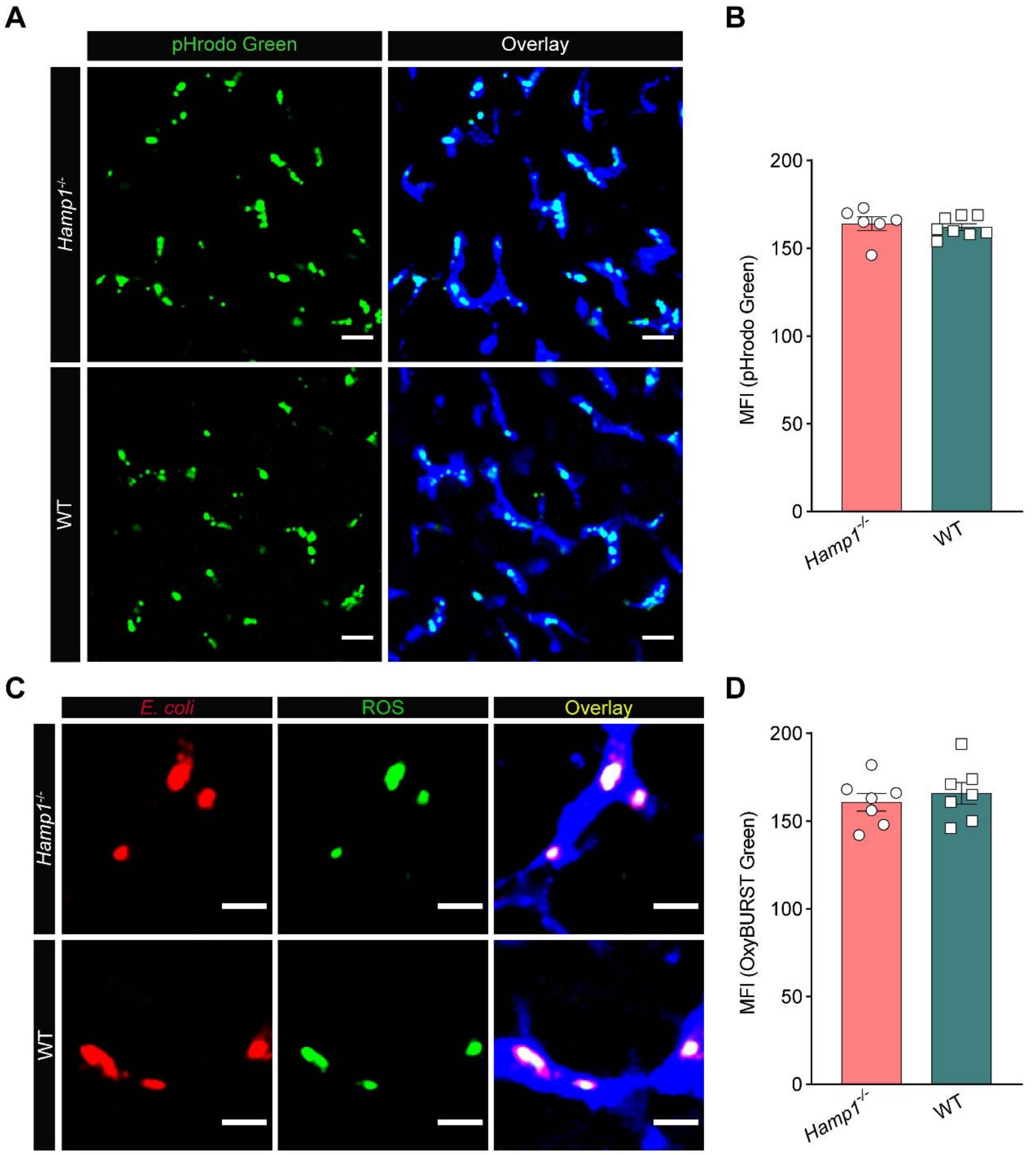

Fig. S6. Assessment of phagosomes acidification and reactive oxygen species generation in KCs. (**A**) Representative IVM images showing KCs (blue) phagocytosed pHrodo Green *E. coli* BioParticles (green) in *Hamp1*$^{-/-}$ and WT mice. Scale bars, 20 μm. (**B**) MFI of pHrodo Green *E. coli* BioParticles uptake by KCs. n = 6-8 per group, data are presented as mean ± SEM. (**C**) Representative IVM images showing ROS-positive (green) Alexa Fluor 555-labelled *E. coli* (red) in KCs (blue) in *Hamp1*$^{-/-}$ and WT mice. Scale bars, 10 μm. (**D**) MFI of OxyBURST Green BioParticles uptake by KCs. n = 7 per group, data are presented as mean ± SEM. By two-tailed Student's *t* test. Data presented are from 6 independent experiments. Each symbol represents an individual mouse. MFI, mean fluorescence intensity; *E. coli*, *Escherichia coli*; ROS, reactive oxygen species; KC, Kupffer cell.

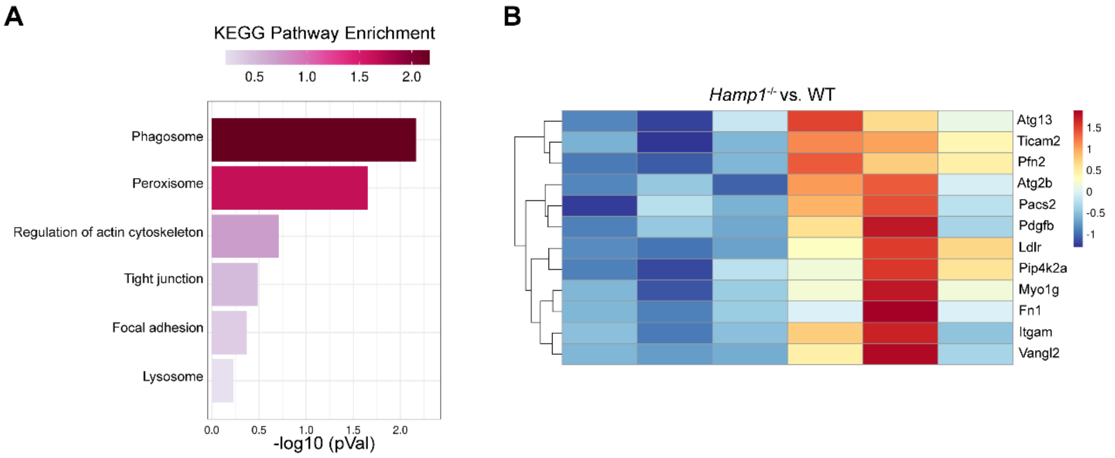

Fig. S7. RNA sequencing analysis of KCs from *Hamp1$^{-/-}$* and WT mice. (**A**) Kyoto Encyclopedia of Genes and Genomes (KEGG) pathway enrichment analysis. (**B**) Heatmap of genes related to the biological processes involved in phagocytosis. n = 3 per group. KC, Kupffer cell.

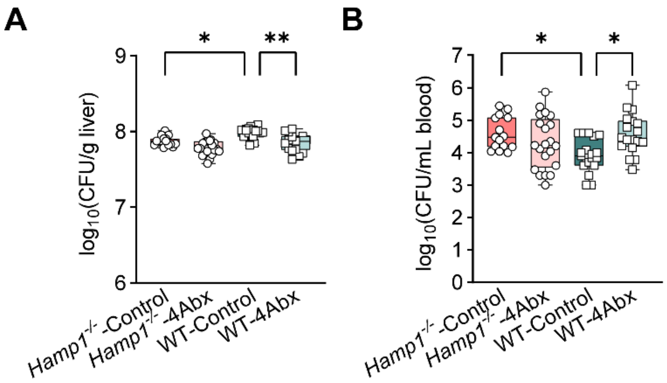

Fig. S8. Gut microbiota depletion impairs hepatic bacterial capture in WT mice. (**A** and **B**) Bacterial load in the livers (**A**) and peripheral blood (**B**) at 2 h after *E. coli* infection in *Hamp1*$^{-/-}$ and WT mice treated with antibiotic cocktail (4Abx) or sterile water (Control). n = 15-21 per group, data are presented as median ± interquartile range. *$p < 0.05$, **$p < 0.01$, by Kruskal–Wallis followed by Dunn's multiple-comparison test. Data presented are from 12 independent experiments. Each symbol represents an individual mouse. *E. coli*, *Escherichia coli*; CFU, colony forming units.

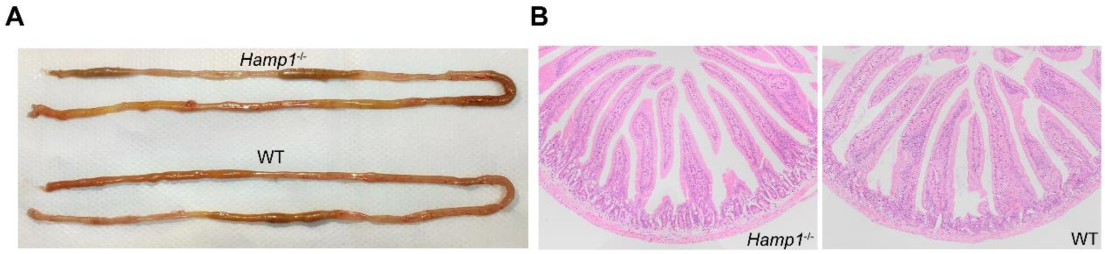

Fig. S9. Anatomical overview and histopathological analysis of small intestine tissues from *Hamp1$^{-/-}$* and WT mice. (**A**) Anatomical overview of small intestine from *Hamp1$^{-/-}$* and WT mice. (**B**) Representative images of small intestine tissues with H&E staining. H&E, hematoxylin and eosin.

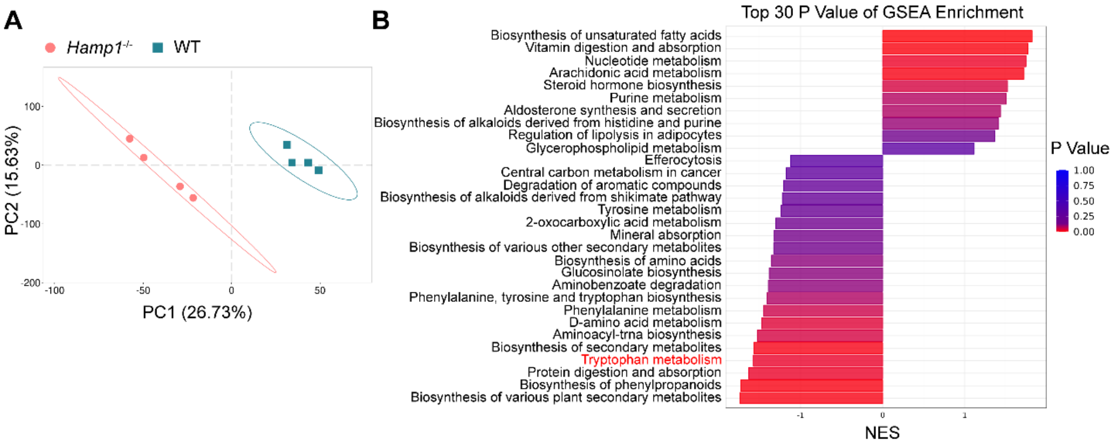

Fig. S10. Hepcidin deficiency remaps portal blood metabolite profiles in mice. (**A**) Partial least squares-discriminant analysis (PLS-DA) based on untargeted metabolomics of portal blood from *Hamp1*$^{-/-}$ and WT mice. n = 4 per group. (**B**) Gene Set Enrichment Analysis (GSEA) showing metabolite sets (top 30) enriched in WT mice compared with *Hamp1*$^{-/-}$ mice. n = 4 per group.

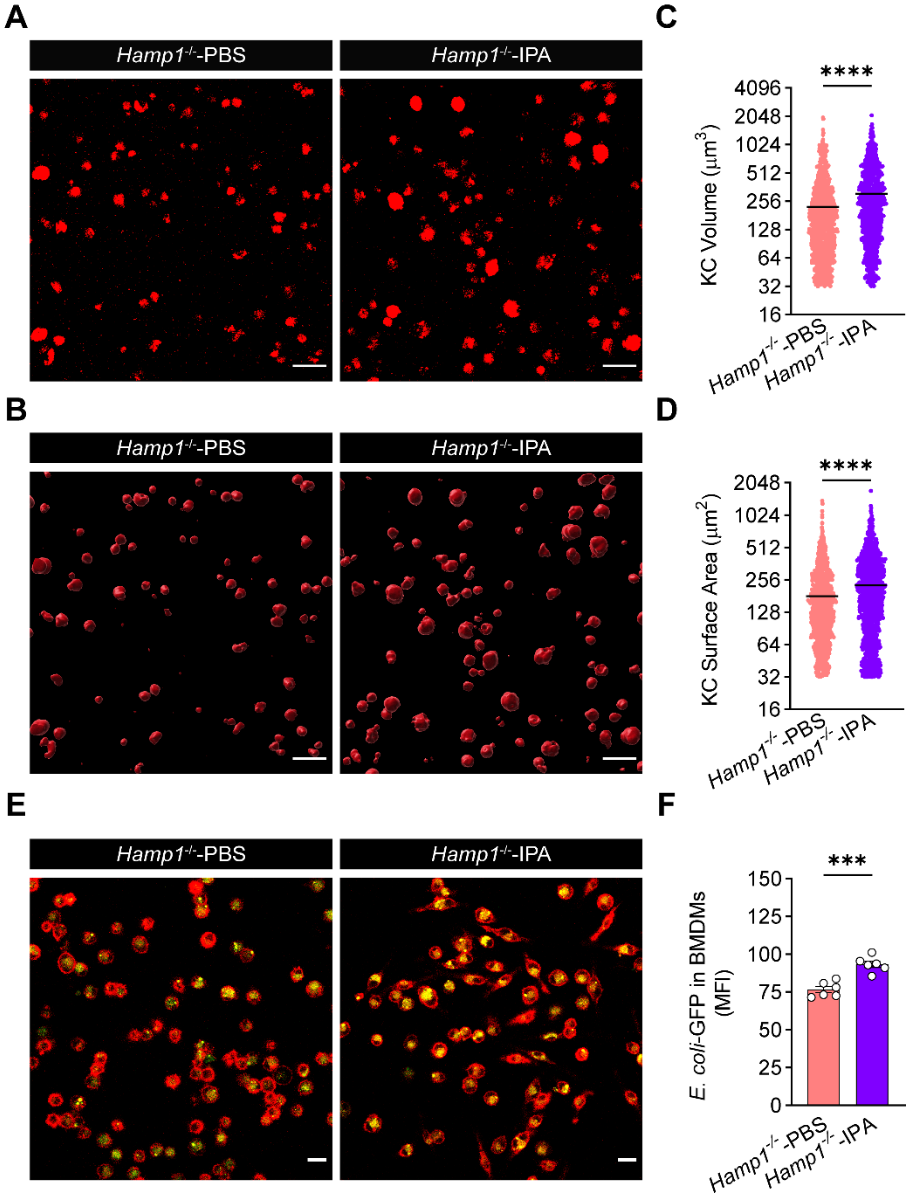

Fig. S11. IPA improves cellular morphology of KCs and phagocytic activity of BMDMs from *Hamp1*$^{-/-}$ mice. (**A**–**D**) IVM image (**A**) combined with three-dimensional reconstruction (**B**) to analyze volume (**C**) and surface area (**D**) of KCs sorted from *Hamp1*$^{-/-}$ mice and pretreated with IPA or sterile PBS. Scale bars, 20 μm. n = 3 per group, data are presented as mean ± SEM. (**E**) Representative images showing phagocytosis of *E. coli*-GFP (yellow) by BMDMs (red) isolated from *Hamp1*$^{-/-}$ mice and pretreated with IPA or sterile PBS. Scale bars, 20 μm. (**F**) MFI of *E. coli*-GFP engulfed by BMDMs. n = 6 per group, data are presented as mean ± SEM. ***$p < 0.001$, ****$p < 0.0001$, by two-tailed Student's *t* test. Data presented are from 3 (A-D) and 6 (E-F) independent experiments. IPA, indole-3-propionic acid; PBS, phosphate-buffered saline; KC, Kupffer cell. BMDMs, bone marrow-derived macrophages; *E. coli*, *Escherichia coli*; GFP, green fluorescent protein; MFI, mean fluorescence intensity.

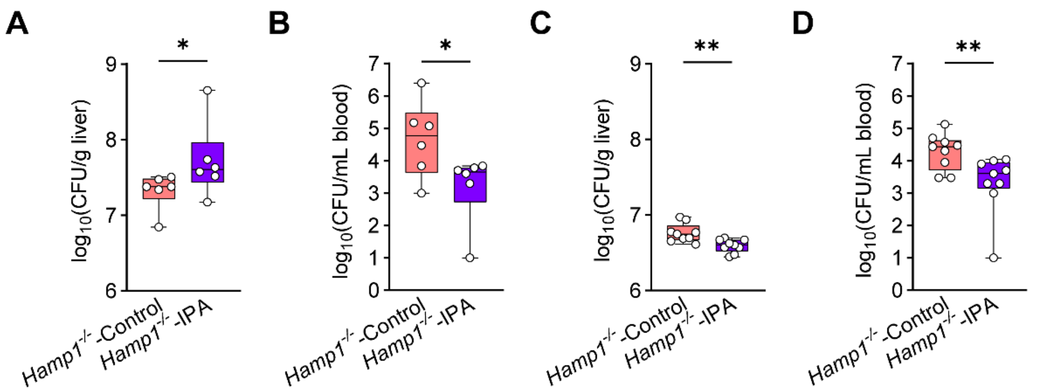

Fig. S12. IPA supplementation restores hepatic immune defense against bloodstream bacterial infection in *Hamp1*$^{-/-}$ mice. (**A** and **B**) Bacterial load in the livers (**A**) and peripheral blood (**B**) at 2 h after *E.* coli infection in *Hamp1*$^{-/-}$ mice pretreated with IPA or sterile PBS (Control). n = 6 per group, data are presented as median ± interquartile range. (**C** and **D**) Bacterial load in the livers (**C**) and peripheral blood (**D**) at 24 h after *E. coli* infection in *Hamp1*$^{-/-}$ mice pretreated with IPA or sterile PBS (Control). n = 9 per group, data are presented as median ± interquartile range. *$p < 0.05$, **$p < 0.01$, by Mann–Whitney *U* test. Data presented are from 5 (A-B) and 4 (C-D) independent experiments. Each symbol represents an individual mouse. IPA, indole-3-propionic acid; *E. coli*, *Escherichia coli*; PBS, phosphate-buffered saline; CFU, colony forming units.

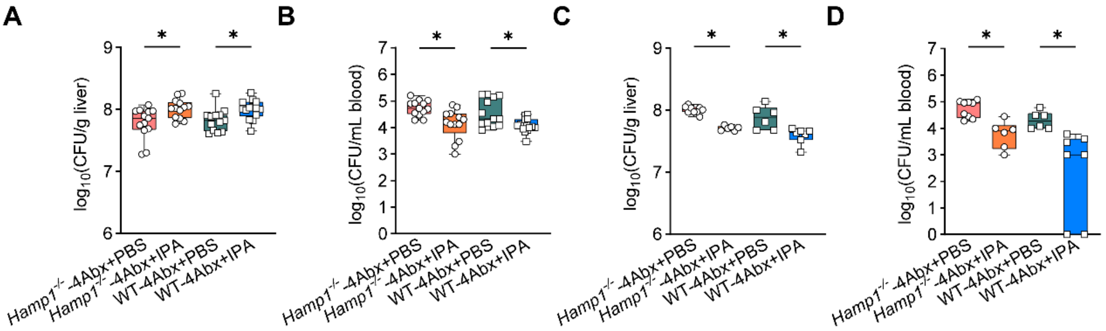

Fig. S13. IPA supplementation improves hepatic antibacterial defense in antibiotics-treated *Hamp1*$^{-/-}$ and WT mice. (**A** and **B**) Bacterial load in the livers (**A**) and peripheral blood (**B**) at 2 h after *E. coli* infection in mice pretreated with antibiotic cocktail (4Abx) and subsequently treated with IPA or sterile PBS. n = 13-14 per group, data are presented as median ± interquartile range. (**C** and **D**) Bacterial load in the livers (**C**) and peripheral blood (**D**) at 24 h after *E. coli* infection in mice pretreated with antibiotic cocktail (4Abx) and subsequently treated with IPA or sterile PBS. n = 6-9 per group, data are presented as median ± interquartile range. *$p < 0.05$, by Kruskal–Wallis followed by Dunn's multiple-comparison test. Data presented are from 4 independent experiments. Each symbol represents an individual mouse. IPA, indole-3-propionic acid; *E. coli*, *Escherichia coli*; PBS, phosphate-buffered saline; CFU, colony forming units.

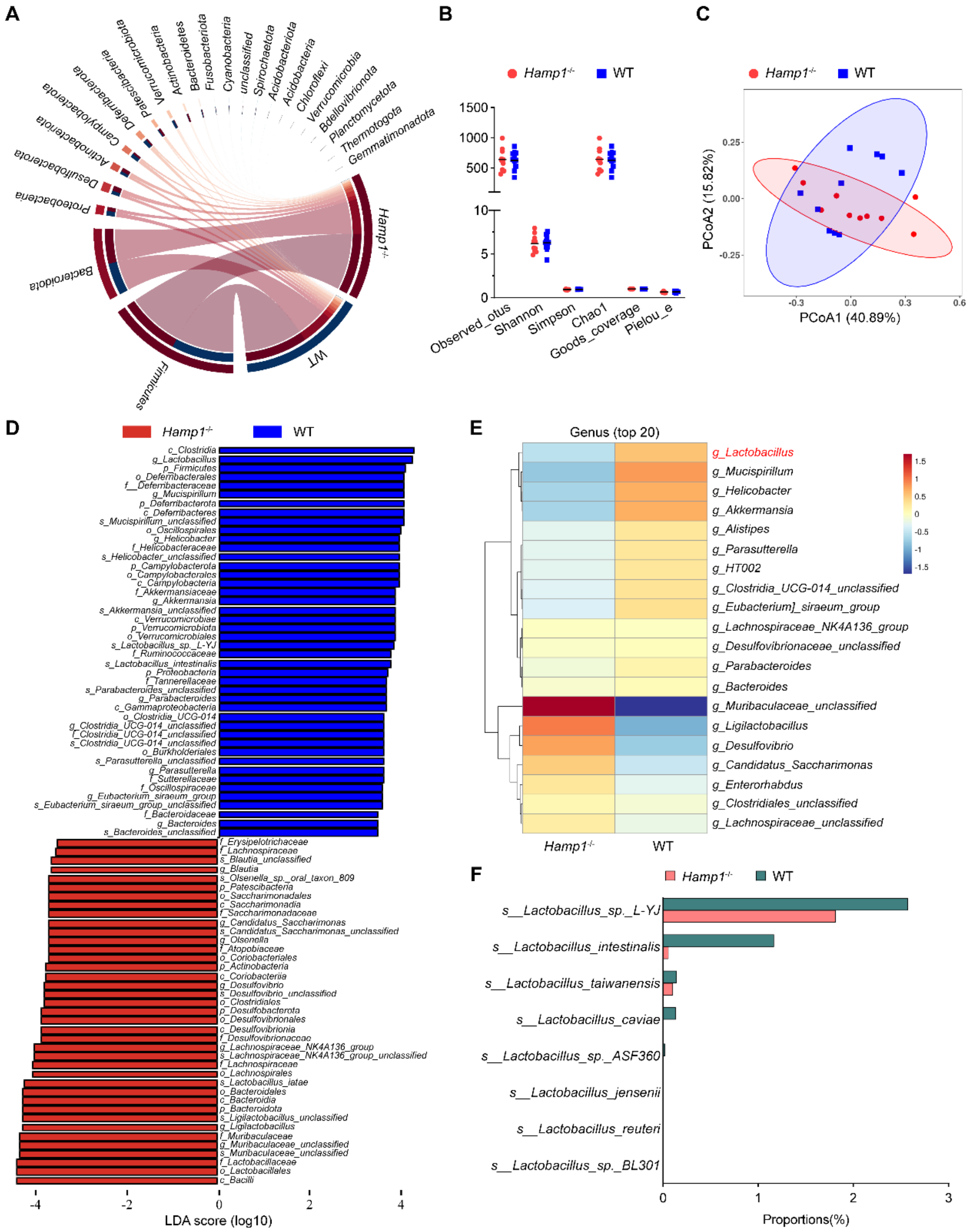

Fig. S14. Hepcidin deficiency alters the abundance of probiotic taxonomic in mouse intestine. (**A**) Circos plot showing gut microbiota composition at the phylum level between *Hamp1*$^{-/-}$ and WT mice. n = 10 per group. (**B**) α-diversity analysis (as accessed by the Observed Species, Shannon, Simpson, Chao1, Good coverage and Pielou e indexes) of gut microbiota composition in *Hamp1*$^{-/-}$ and WT mice. n = 10 per group, data are presented as scatter plot with mean. (**C**) Scatter plot of principal coordinate analysis (PCoA) showing β-diversity analysis of gut microbiota composition in *Hamp1*$^{-/-}$ and WT mice. n = 10 per group. (**D**) Linear discriminant analysis effect size (LEfSe) assessing taxonomic differences between *Hamp1*$^{-/-}$ and WT mice. Linear discriminant analysis (LDA) > 3.5. n = 10 per group. (**E**) Heatmap showing the enrichment of the top 20 genus in the gut microbiota composition of *Hamp1*$^{-/-}$ and WT mice. n = 10 per group. (**F**) Bar plot of 16S rDNA sequencing of fecal samples from *Hamp1*$^{-/-}$ and WT mice at the species level. n = 10 per group.

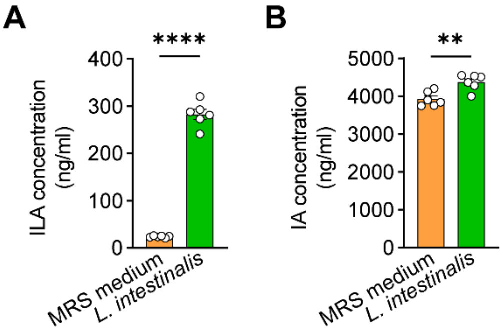

Fig. S15. ILA and IA concentration in the culture supernatant of *L. intestinalis* and MRS medium. (**A-B**) ILA **(A)** and IA **(B)** concentration in the culture supernatant of *L. intestinalis* and MRS medium. n = 6 per group, data are presented as mean ± SEM. **p < 0.01, ****p < 0.0001, by two-tailed Student's *t* test. Data presented are from 6 (A-B) independent experiments. ILA, indole-3-lactic acid; IA, indole-3-acrylic acid; *L. intestinalis*, *Lactobacillus intestinalis*; MRS, Man, Rogosa and Sharpe.

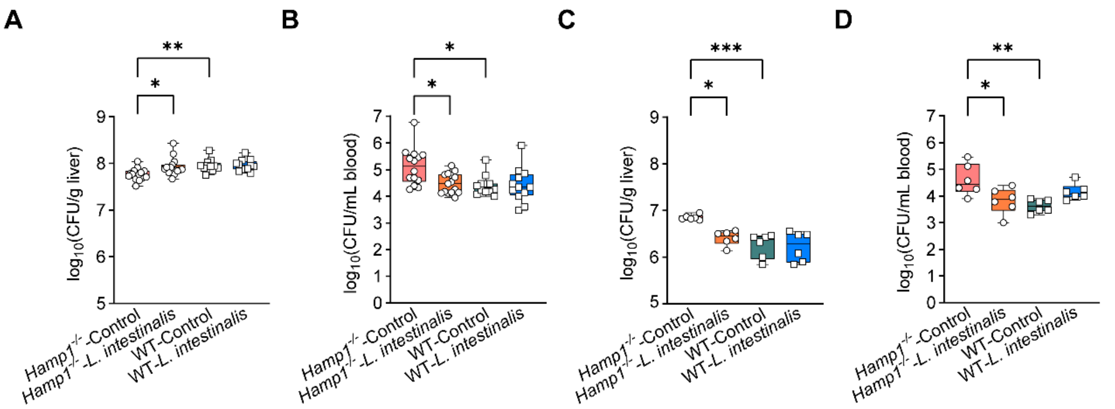

Fig. S16. Colonization by IPA-producing *L. intestinalis* restores hepatic immune defense against bloodstream bacterial infection in *Hamp1*$^{-/-}$ mice. (**A** and **B**) Bacterial load in the livers (**A**) and peripheral blood (**B**) at 2 h after *E. coli* infection in mice pretreated with *L. intestinalis* or sterile PBS (Control). n = 12-15 per group, data are presented as median ± interquartile range. (**C** and **D**) Bacterial load in the livers (**C**) and peripheral blood (**D**) at 24 h after *E. coli* infection in mice pretreated with *L. intestinalis* or sterile PBS (Control). n = 6 per group, data are presented as median ± interquartile range. **p* < 0.05, ***p* < 0.01, ****p* < 0.001, by Kruskal–Wallis followed by Dunn's multiple-comparison test. Data presented are from 10 (A-B) and 4 (C-D) independent experiments. Each symbol represents an individual mouse. *L. intestinalis*, *Lactobacillus intestinalis*; *E. coli*, *Escherichia coli*; PBS, phosphate-buffered saline; CFU, colony forming units.

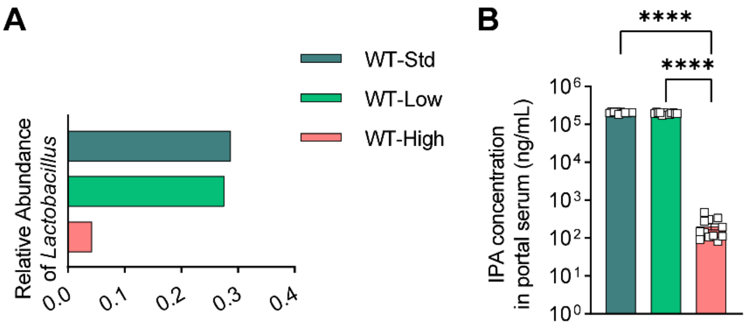

Fig. S17. Dietary iron manipulation affects *Lactobacillus* abundance and IPA production in WT mice. (**A**) Bar plot of 16S rDNA sequencing of fecal samples from WT mice treated with a standard diet (WT-Std), a low-iron diet (WT-Low) or with a high-iron diet (WT-High) at the genus level. n = 11-13 per group. (**B**) Portal blood level of IPA in WT-Std, WT-Low and WT-High mice. n = 11-13 per group, data are presented as mean ± SEM. ****$p < 0.0001$, by one-way ANOVA followed by Šidák's multiple-comparison test (B). Data presented are from 4 (A-B) independent experiments. Each symbol represents an individual mouse. IPA, indole-3-propionic acid.

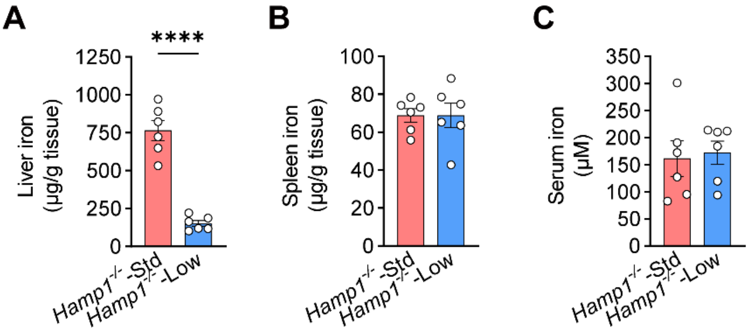

Fig. S18. Iron load in *Hamp1$^{-/-}$* mice after iron depletion. (**A-C**) Liver iron (**A**), spleen iron (**B**) and serum iron (**C**) in *Hamp1*$^{-/-}$ mice treated with a standard diet (*Hamp1*$^{-/-}$-Std) or with a low-iron diet (*Hamp1*$^{-/-}$-Low). n = 6 per group, data are presented as mean ± SEM. ****$p < 0.0001$, by two-tailed Student's t test. Data presented are from 2 independent experiments. Each symbol represents an individual mouse.

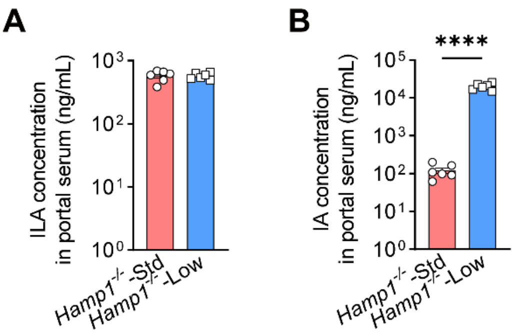

Fig. S19. Portal blood levels of ILA and IA in *Hamp1*$^{-/-}$ mice fed with low-iron diet. (**A-B**) Portal blood levels of ILA **(A)** and IA **(B)** in *Hamp1*$^{-/-}$-Std and *Hamp1*$^{-/-}$-Low mice. n = 6 per group, data are presented as mean ± SEM. ****p < 0.0001, by two-tailed Student's t test. Data presented are from 2 independent experiments. Each symbol represents an individual mouse (A -B). ILA, indole-3-lactic acid; IA, indole-3-acrylic acid.

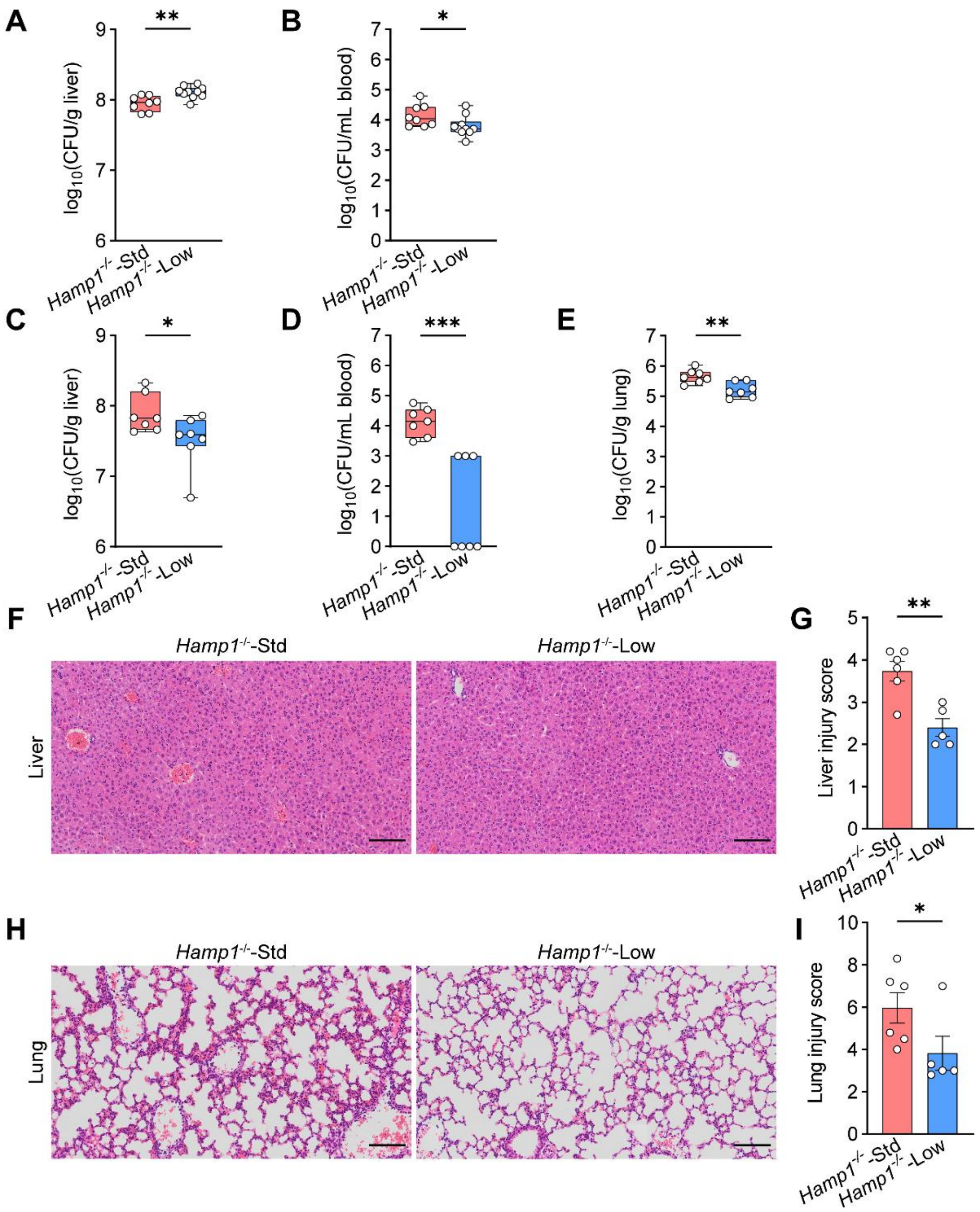

Fig. S20. Dietary iron restriction improves KC bacterial clearance and liver immune defense in *Hamp1$^{-/-}$* mice. (**A** and **B**) Bacterial load in the in the livers (**A**) and peripheral blood (**B**) at 2 h after *E. coli* infection in *Hamp1$^{-/-}$* mice treated with a standard diet (*Hamp1$^{-/-}$*-Std) or with a low-iron diet (*Hamp1$^{-/-}$*-Low). n = 8-10 per group, data are presented as median ± interquartile range. (**C**–**E**) Bacterial load in the in the livers (**C**), peripheral blood (**D**) and lungs (**E**) at 24 h after *E. coli* infection in *Hamp1$^{-/-}$*-Std and *Hamp1$^{-/-}$*-Low mice. n = 7 per group, data are presented as median ± interquartile range. (**F**) Representative images of livers with H&E staining at 24 h after *E. coli* infection in *Hamp1$^{-/-}$*-Std and *Hamp1$^{-/-}$*-Low mice. Scale bar, 50 µm. (**G**) Quantification of liver injury score. n = 5-6 per group, data are presented as mean ± SEM. (**H**) Representative images of lungs with H&E staining at 24 h after *E. coli* infection in *Hamp1$^{-/-}$*-Std and *Hamp1$^{-/-}$*-Low mice. Scale bar, 50 µm. (**I**) Quantification of lung injury score. n = 5-6 per group, data are presented as mean ± SEM. *$p < 0.05$, **$p < 0.01$, ***$p < 0.001$, by Mann–Whitney *U* test (A-B, C-E), two-tailed Student's *t* test (F-I). Data presented are from 4 independent experiments. Each symbol represents an individual mouse. *E. coli*, *Escherichia coli*; H&E, hematoxylin and eosin; CFU, colony forming units.

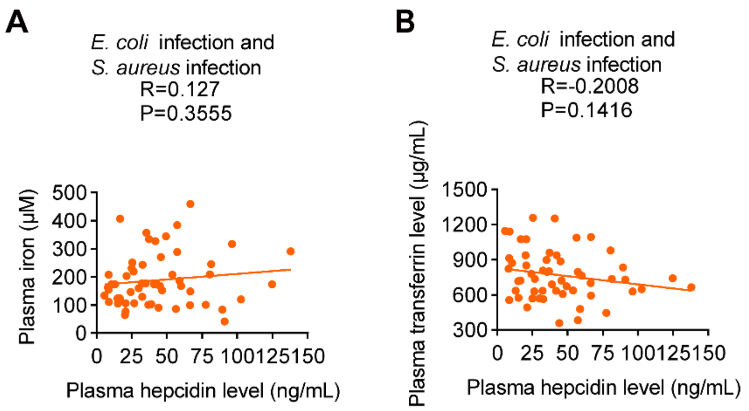

Fig. S21. Correlation of plasma iron and plasma transferrin level with plasma hepcidin level in patients with bacteremia. (**A**) Correlation of plasma iron with plasma hepcidin level in all patients with bacteremia (n = 55). (**B**) Correlation of plasma transferrin level with plasma hepcidin level in all patients with bacteremia (n = 55). By Pearson's correlation analysis. *E. coli*, *Escherichia coli*; *S. aureus*, *Staphylococcus aureus*.

Table S1. Demographic and clinical characteristics of the study cohort.

Characteristics	Non-infection (n =25)	*S. aureus* infection (n = 28)	*E. coli* infection (n = 27)
Male sex, n (%)	15 (60.0%)	19 (67.9%)	19 (70.4%)
Age (months)	25.64 (±39.83)	40.39 (±54.55)	14.89 (±35.74)
Weight (kg)	9.93 (±9.51)	14.92 (±15.07)	8.35 (±10.87)
Hepcidin (ng/mL)	17.38 (±13.78)	42.12 (±25.06)^{A}	46.33 (±34.92)^{B}
Iron (μM)	-	192.9 (±104.4)	184.2 (±83.95)
Ferritin (ng/mL)	-	146.9 (±132.8)	188.2 (±176.7)
Transferrin (μg/mL)	-	771.0 (±200.7)	765.1 (±222.9)
WBC (×10^9/L)	9.523 (±3.541)	10.69 (±6.06)	12 (±11.9)
SCRP (mg/L)	14.68 (±27.67)	39.22 (±36.24)^{C}	48.49 (±56.98)^{A}
Platelets (×10^3/μL)	376.0 (±130.3)	276.0 (±160.5)^{A}	324.1 (±167.5)
Antibiotic usage (days)	5.44 (±6.178)	25.36 (±25.73)^{C}	15.85 (±15.48)^{B}
ICU stay (days)	1.24 (±4.206)	6.393 (±19.77)	5.296 (±16.98)
Hospital stay (days)	10.88 (±9.897)	32.43 (±29.89)^{B}	21 (±25.24)

Data are presented as mean ± SD, Fisher's exact test or Mann-Whitney U test was performed for comparison between two experimental groups. WBC, white blood cell; SCRP, supersensitive C-reactive protein; ICU, intensive care unit. $^{A}p < 0.01$. $^{B}p < 0.001$. $^{C}p < 0.0001$. $^{A, B, C}$Compared with non-infection group.

Table S2. Antibodies or Resources.

Antibodies or Resources	Company	Identifier
**Antibodies**		
Brilliant Violet 421-conjugated F4/80	BioLegend	Cat# 123132; RRID: AB_10901171
Alexa Fluor 700 CD45 Antibody	BioLegend	Cat# 103128; RRID: AB_493714
PE F4/80 Antibody	BioLegend	Cat# 123109; RRID: AB_893486
PerCP/Cyanine5.5 CD11b Antibody	BioLegend	Cat# 101227; RRID: AB_893232
FITC anti-CD11b (M1/70)	eBioscience	Cat# 11-0112-82; RRID: AB_464935
Brilliant Violet 650 MHC II Antibody	BioLegend	Cat# 107641; RRID: AB_2565975
PE/Cyanine7 Tim-4 Antibody	BioLegend	Cat# 130009; RRID: AB_2565718
APC ESAM Antibody	BioLegend	Cat# 136207; RRID: AB_2101658
Brilliant Violet 785 CD206 Antibody	BioLegend	Cat# 141729; RRID: AB_2565823
F4/80 Monoclonal Antibody (Cl: A3-1)	Fisher Scientific	Cat# MA5-16630; RRID: AB_2538126
Donkey anti-Rat IgG (H+L) Highly Cross-Adsorbed Secondary Antibody, Alexa Fluor Plus 647	Fisher Scientific	Cat# A48272TR; RRID: AB_2896338
**Bacterial strains**		
Escherichia coli	ATCC	ATCC: 25922
Escherichia coli-GFP	ATCC	ATCC: 25922GFP
Staphylococcus aureus	ATCC	ATCC: 43300
Staphylococcus aureus-GFP	BIO SCI	RN4220::sfGFP
Lactobacillus intestinalis	DSMZ	DSM 6629
**Chemicals**		
Clophosome-Clodronate Liposomes Plain control Liposomes (Neutral)	FormuMax	Cat# F70101C-NC-10
Collagenase, Type I, powder	Gibco	Cat# 17018029
HEPES	Sigma-Aldrich	Cat# V900477
Sodium chloride	Sinopharm Chemical Reagent	Cat# 10019318
Potassium chloride	Sinopharm Chemical Reagent	Cat# 10016318
Sodium hydrogen carbonate	Sinopharm Chemical Reagent	Cat# 10018960
D-(+)-Glucose monohydrate	Sinopharm Chemical Reagent	Cat# 10010518
Ethylenediamine tetraacetic acid	Sinopharm Chemical Reagent	Cat# 10009617

Agar-agar power	Sinopharm Chemical Reagent	Cat# 10000561
YEAST EXTRACT	Fisher Scientific	Cat# LP0021B
TRYPTONE	Fisher Scientific	Cat# LP0042B
Phosphate buffered solution (PBS), 1X	Yuchun Biology	Cat# YC-5013
4% Paraformaldehyde Solution	biosharp	Cat# BL539A
Tissue-Tek O.C.T. Compound	SAKURA	Cat# 4583
Tween-20	Sigma-Aldrich	Cat# P7949
Triton X-100	Sigma-Aldrich	Cat# X100
Bovine Serum Albumin (BSA)	Sigma-Aldrich	Cat# A8022
pHrodo™ Green *E. coli* BioParticles™	Fisher Scientific	Cat# P35366
OxyBURST™ Green H2DCFDA, SE	Fisher Scientific	Cat# D2935
Alexa Fluor™555 NHS	Fisher Scientific	Cat# A37571
Hydroxylamine hydrochloride	Sigma-Aldrich	Cat# 159417
Antifade Mounting Medium with DAPI	Beyotime	Cat# P0131
Vancomycin hydrochloride	MCE	Cat# HY-17362
Ampicillin	Sigma-Aldrich	Cat# A5354
Metronidazole	Sigma-Aldrich	Cat# M1547
Neomycin trisulfate salt hydrate	Sigma-Aldrich	Cat# N6386
DMEM, high glucose, pyruvate	Gibco	Cat# 11995065
DAPI dihydrochloride	Beyotime	Cat# C1002
Fetal Bovine Serum	Biological Industries	Cat# 01-100-1A
Penicillin-Streptomycin Liquid (100 X)	Solarbio	Cat# P1400
Indole-3-propionic acid (IPA)	Aladdin	Cat# I103959
de Man, Rogosa and Sharpe	MINGZHOUBIO	Cat# MZM1549
Standard diet	Xietong	Cat# XT93G
Low-iron diet	Xietong	Cat# XTLFe01
High-iron diet	Xietong	Cat# XTMM
**Commercial assays**		
Hepcidin 25 (Bioactive) HS ELISA Kit	DRG	Cat# EIA-5782
Non-heme iron assay kit	LEAGENE	Cat# TC1000
Human Ferritin ELISA Kit	abcam	Cat# ab108837
Human Transferrin ELISA Kit	abcam	Cat# ab187391
**Software**		
FlowJo (v10.6.2)	Becton, Dickinson and Company	https://www.flowjo.com/
Prism 9.5.0	GraphPad	https://www.graphpad.com/scientific-software/prism/
ImageJ	NIH	https://imagej.nih.gov/ij/
Imaris	OXFORD	https://imaris.oxinst.com/
